# Identifying noncoding risk variants using disease-relevant gene regulatory networks

**DOI:** 10.1038/s41467-018-03133-y

**Published:** 2018-02-16

**Authors:** Long Gao, Yasin Uzun, Peng Gao, Bing He, Xiaoke Ma, Jiahui Wang, Shizhong Han, Kai Tan

**Affiliations:** 1 Department of Genetics, Perelman School of Medicine, University of Pennsylvania, Philadelphia, PA 19104 USA; 2 Division of Oncology and Center for Childhood Cancer Research, Children’s Hospital of Philadelphia, Philadelphia, PA 19104 USA; 3 Department of Biomedical and Health Informatics, Children’s Hospital of Philadelphia, Philadelphia, PA 19104 USA; 4 School of Computer Science and Technology, Xidian University, Xi’an, 710126 Shaanxi China; 5 The Jackson Laboratory, Farmington, CT 06032 USA; 6 Department of Psychiatry and Behavioral Sciences, Johns Hopkins University School of Medicine, Baltimore, MD 21287 USA; 7 Department of Pediatrics, Perelman School of Medicine, University of Pennsylvania, Philadelphia, PA 19104 USA; 8 Department of Cell & Developmental Biology, Perelman School of Medicine, University of Pennsylvania, Philadelphia, PA 19104 USA

## Abstract

Identifying noncoding risk variants remains a challenging task. Because noncoding variants exert their effects in the context of a gene regulatory network (GRN), we hypothesize that explicit use of disease-relevant GRNs can significantly improve the inference accuracy of noncoding risk variants. We describe Annotation of Regulatory Variants using Integrated Networks (ARVIN), a general computational framework for predicting causal noncoding variants. It employs a set of novel regulatory network-based features, combined with sequence-based features to infer noncoding risk variants. Using known causal variants in gene promoters and enhancers in a number of diseases, we show ARVIN outperforms state-of-the-art methods that use sequence-based features alone. Additional experimental validation using reporter assay further demonstrates the accuracy of ARVIN. Application of ARVIN to seven autoimmune diseases provides a holistic view of the gene subnetwork perturbed by the combinatorial action of the entire set of risk noncoding mutations.

## Abstract (web summary)

Current methods for prioritization of non-coding genetic risk variants are based on sequence and chromatin features. Here, Gao et al. develop ARVIN, which predicts causal regulatory variants using disease-relevant gene-regulatory networks, and validate this approach in reporter gene assays.

## Introduction

Genome-wide association studies (GWASs) and whole-genome sequencing have revealed thousands of sequence variants associated with different human diseases/traits^1–3^. The vast majority of identified variants are located outside of coding sequences, making direct interpretation of their functional effects challenging. For the small number of cases where the causal variants have been experimentally validated, they have been shown to perturb binding sites of transcription factors, local chromatin structure or co-factor recruitment, ultimately resulting in changes of transcriptional output of the target gene(s)^4–6^.

Among the different classes of noncoding regulatory sequences, transcriptional enhancers represent the primary basis for differential gene expression, with many human diseases resulting from altered enhancer action^5,7,8^. Numerous recent studies have uncovered a large number of putative enhancers in a diverse array of human cells and tissues^9–11^. Overlapping the catalog of genetic variants with known enhancers has revealed an enrichment of disease-associated variants in tissue-specific enhancers^12,13^, emphasizing the importance of knowledge about tissue-specific *cis*-regulatory sequences for identifying causal variants. In the following, we term single nucleotide polymorphisms (SNPs) located in enhancers eSNPs. A number of computational methods have been developed to predict causal noncoding variants^14–20^. Conceptually, these methods operate by annotating genetic variants using a catalog of *cis*-regulatory sequences (based on chromatin accessibility, transcription factor binding, epigenetic modification signatures). Although biologically intuitive, such an approach does not take into account the complex interactions of the underlying gene regulatory network (GRN) in which a causal noncoding variant exerts its effect, namely, interactions among transcription factors and their target genes as well as interactions among target genes in the same pathway. Molecular networks have been explicitly used to improve the inference accuracy of causal coding variants^21–24^. This potential has not been examined for noncoding variants. To address these shortcomings, we postulate that (1) the impact of causal eSNPs on gene expression is transmitted through the GRNs in the cell/tissue types that are relevant to the studied trait; and (2) the genes affected by the full set of causal eSNPs for a trait are organized in a limited number of pathways. We test this hypothesis by developing a general computational framework for identifying causal noncoding variants that affect a specific disease/trait.

Linkage disequilibrium (LD) presents another challenge for finding causal noncoding variants. By casting the causal inference problem into a subnetwork identification problem, our method evaluates both GWAS lead SNPs and linked SNPs simultaneously, thus increasing the power of the inference. Further, our network-based approach naturally provides a pathway content for understanding the predicted causal eSNPs.

We characterize the performance of our method using known risk mutations in gene promoters in 20 diseases and gene enhancers in 10 diseases. We further validate randomly selected predictions using luciferase reporter assay. By applying our method to seven autoimmune diseases, we obtain a systems view of the entire set of risk eSNPs in a given disease and equally important the subnetwork that is perturbed by the set of risk eSNPs.

## Results

### Construction of disease-relevant gene regulatory network

**Fig. 1:**
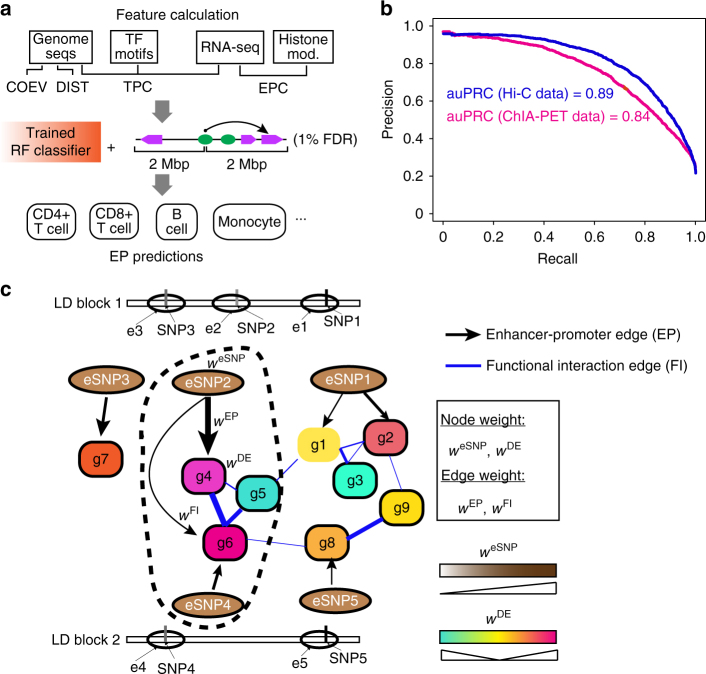
Construction of weighted and disease-relevant gene regulatory network for prioritizing risk SNPs located in regulatory DNA sequences. **a** The IM-PET algorithm for predicting enhancer targets. Features used by the random forest (RF) classifier are: COEV, coevolution of enhancer and target promoter; DIST, distance constraint between enhancer and target promoter; TPC, transcription factor and target promoter correlation; EPC, enhancer and target promoter profile correlation; FDR, false discovery rate. **b** Performance evaluation of IM-PET using Hi-C and ChIA-PET data. Sources of Hi-C and ChIA-PET data are listed in Supplementary Table 2. **c** Schematic for an integrated, disease-relevant gene regulatory network. The network involves SNP-containing enhancers and their target genes and functional interactions among the target genes. Such a network is constructed by integrating transcriptomic and epigenomic data on cells/tissues relevant to the disease under study. The encircled subnetwork represents pathways targeted by a candidate risk eSNP. LD, linkage disequilibrium; e, enhancer; g, gene; EP, enhancer−promoter interaction; FI, functional gene interaction; eSNP, enhancer SNP; *W*^eSNP^, weights for eSNPs; *W*^DE^, weights for differential gene expression; *W*^EP^, weights for EP edges; *W*^FI^, weights for FI edges

A number of previous studies have reported enrichment of GWAS SNPs in regulatory DNA sequences specific to disease-relevant tissues or cell types^12,13^, emphasizing the importance of knowledge about tissue-specific regulatory sequences for identifying risk variants. Additionally, gene−gene and protein−protein interaction networks have been used to identify causal coding variants^21,25,26^. Because the effects of non-coding variants are transcriptionally integrated, a network-based approach should be an effective strategy to identify causal noncoding variants. To date, tissue-relevant GRN has not been used explicitly to prioritize noncoding variants. As a first step towards this goal, we sought to construct an integrative GRN for each disease-relevant cell/tissue type. We integrated epigenomic, transcriptomic and functional gene−gene interactions to construct the network. Our integrative network has two parts, the first part involves interactions between enhancers and target genes EP edges, which is a major challenge in constructing GRN in general. By using our recently developed algorithm, IM-PET (Fig. 1a)^27^, we constructed 23 cell/tissue-specific enhancer−promoter (EP) networks that are relevant to the set of 16 diseases in this study (Supplementary Table 1). We evaluated the accuracy of IM-PET using a compendium of Hi-C and ChIA-PET chromatin interaction data from nine cell types (GM12878, K562, IMR90, HMEC, NHEK, HUVEC, Hela, CD34+ cells, and CD4+ T cells, Supplementary Table 2). The overall Area Under the Precision and Recall Curve (auPRC) curve were 0.89 and 0.84 using Hi-C and ChIA-PET interactions as the gold standard, respectively (Fig. 1b), suggesting high quality of the EP predictions by IM-PET. The second part of the integrative network consists of functional interactions between target genes. For this, we used probabilistic functional gene interaction network inferred by integrating multiple lines of evidence (i.e. HumanNet, see Methods) ^21^. Interactions in the backbone HumanNet are not disease-specific; to add disease-specific information for the functional gene interaction network, we add differential gene expression information from case vs control comparison in disease-relevant cells/tissues. The resulting integrative GRN contains two types of edges, EP edges representing enhancer−promoter interactions and FI edges representing functional gene−gene interactions (Fig. 1c). The final product is an edge- and node-weighted, disease-relevant GRN, which is used for predicting risk noncoding variants. See Methods for additional details about the network construction.

### ARVIN combines sequence-based and network-based features

**Fig. 2:**
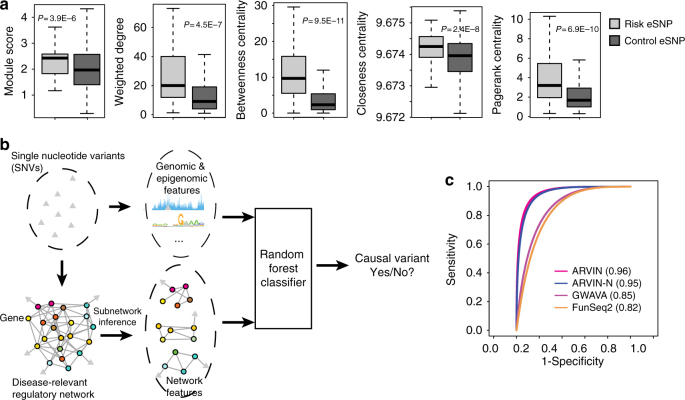
ARVIN combines genomic, epigenomic, and network features to prioritize risk SNPs. **a** Network features extracted from a disease-relevant gene regulatory network are discriminative. *P *values are based on *t *test. **b** Overview of ARVIN. **c** Receiver Operating Characteristic (ROC) curves using known risk SNPs located in gene promoters. Values in parenthesis are area under the ROC curve. *P *values are computed using a bootstrap-based method^55^. ARVIN-N ARVIN using network-based features only

We hypothesized that disease-relevant GRN could improve the inference accuracy of noncoding risk variants. To this end, we examined a number of network-based features to see if they can discriminate true risk SNPs from negative control SNPs. We obtained 233 gold-standard risk SNPs located in gene promoters from the Human Gene Mutation Database (HGMD) ^28^. This set of SNPs is associated with 20 different diseases (Supplementary Data 1). We assigned a *W*^EP^ value of 1 to edges between an SNP and the genes whose promoter harbors the SNP, since the gene promoters are annotated with very high confidence in the Ensembl database. We used gene expression data of case and control samples (Supplementary Table 3) to compute the gene weight, *W*^DE^. Next, we used the constructed disease-relevant GRNs to compute the following network-based features: module score, weighted node degree, betweenness centrality, closeness centrality, and page rank centrality (see Methods for details). These features are designed to evaluate the topological importance of the direct target gene of a promoter or enhancer SNP as well as the local network neighborhood of the target gene. Our hypothesis is that target genes with large topological importance in the GRN might be rate-limiting genes for disease pathogenesis. We found that the set of network features can indeed distinguish true risk SNPs from control SNPs (Fig. 2a). Next, we compared the discriminative power of disease-specific and non-disease-specific networks. We found that values of network features are less separated between risk and control SNPs when using non-disease-specific networks (Supplementary Fig. 1), further supporting utility of disease-specific network for identifying risk SNPs.

To further test the discriminative power of the network-based features, we built a random forest (RF) classifier using these features and sequence-based features used by two state-of-the-art methods, genome-wide annotation of variants (GWAVA)^16^ and FunSeq2^20^. We evaluated the relative importance of all features (six from this study and 182 from GWAVA and FunSeq2 combined) by using a recursive feature elimination (RFE) approach. Applying the RFE procedure yielded a set of 35 most discriminative features based on classification error (Supplementary Figs 2 and 3). Strikingly, all network-based features were ranked in the top ten (Supplementary Data 2), suggesting that network-based features are independently discriminative from the sequence-based features. On the other hand, the fact that 35 features were selected suggests that network-based features and sequence-based features are complementary to each other. We examined potential interactions among selected features and found significant association between network-based features and sequence-based features, further supporting the notion that these two types of features are complementary (Supplementary Fig. 4). Based on this finding, we developed the Annotation of Regulatory Variants using Integrated Networks (ARVIN) algorithm by combining network features with sequence features (Fig. 2b). We evaluated the classification accuracy using fivefold cross- validation and the set of 233 gold-standard risk SNPs in gene promoters. ARVIN achieved an area under the ROC curve (auROC) of 0.96, significantly larger than those of GWAVA (auROC = 0.85, *P* = 1.7×10^−12^) and FunSeq2 (auROC = 0.82, *P* = 4.2×10^−15^) (Fig. 2c).

**Fig. 3:**
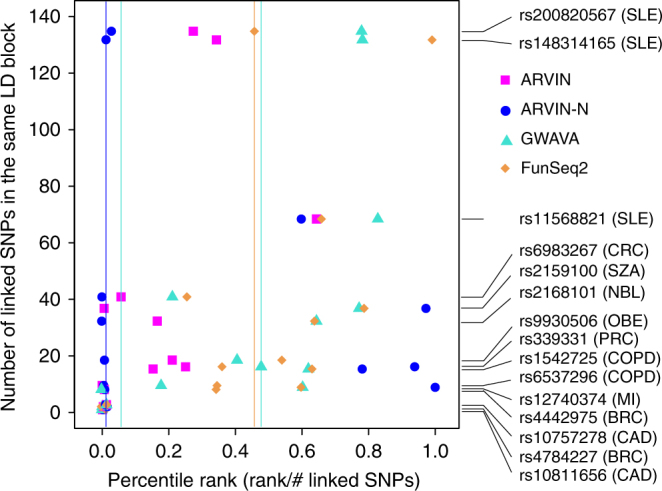
Performance benchmarking using known risk SNPs located in enhancers. References for validated risk enhancer SNPs are provided in Supplementary Table 4. *Y*-axis represents the number of linked eSNPs in the same LD block as the known risk SNP(s). Performance is expressed as percentile ranking on the *x*-axis in which each gold-standard risk SNP was ranked against all other SNPs in the same linkage equilibrium block as the gold-standard SNP. Filled symbols, rank of an individual gold-standard SNP by a given method. Vertical lines, median rank of the full set of gold-standard SNPs by a given method. SNP IDs and associated diseases are shown on the right. SLE, systemic lupus erythematosus; PSO, psoriasis; CRC, colorectal cancer; PRC, prostate cancer; RA, rheumatoid arthritis; OBE, obesity; MI, myocardial infarction; BRC, breast cancer; COPD, chronic obstructive pulmonary disease; SZA, schizophrenia; CAD, coronary artery disease; NBL, neuroblastoma

Many genes are regulated by distal enhancers. Compared to promoter variants, risk variants located in distal enhancers are more challenging to study due to the difficulty of assigning enhancer targets and existence of multiple enhancers targeting the same gene. We further tested the performance of ARVIN using risk SNPs located in enhancers. We curated a set of 15 experimentally validated risk enhancer SNPs implicated in ten complex diseases, including autoimmune, heart, lung, psychiatric diseases, obesity, and cancer (Supplementary Table 4). Compared to promoter variants, the set of gold-standard enhancer variants is too small for ROC curve analysis to be meaningful. Therefore, for each risk SNP, we asked how it is ranked by a method among all enhancer SNPs in the same LD block as the risk SNP. The number of linked eSNPs ranges from 1 to 168 with an average of 28 (Supplementary Table 6), highlighting the difficulty of identifying true risk SNPs. Overall, both ARVIN and ARVIN with network feature alone (ARVIN-N) outperformed GWAVA and FunSeq2. The median percentile ranking of the set of known risk eSNPs were 1, 5, 47, and 45% for ARVIN-N, ARVIN, GWAVA, and FunSeq2, respectively (vertical lines, Fig. 3).

In summary, using gold-standard risk SNPs in both promoters and enhancers, we demonstrate that incorporation of network features can significantly improve the accuracy of finding risk enhancer SNPs.

### Application of ARVIN to autoimmune diseases

We applied ARVIN to identify risk eSNPs associated with seven autoimmune diseases (Crohn’s disease, multiple sclerosis, psoriasis, rheumatoid arthritis, systemic lupus erythematosus, type 1 diabetes, and ulcerative colitis). We first obtained lead SNPs associated with those diseases from the National Human Genome Research Institute (NHGRI) GWAS Catalog^29^. On average, there are 123 GWAS lead SNPs per disease (Supplementary Table 5). As candidate SNPs, we considered both lead SNPs and SNPs that are in the same LD block with the lead SNPs. By overlapping SNPs with enhancers from disease-relevant cell/tissue types, we obtained the list of eSNPs as the final input to ARVIN. On average, there are 66 eSNPs for each disease-associated locus tagged by a lead GWAS SNP.

**Fig. 4:**
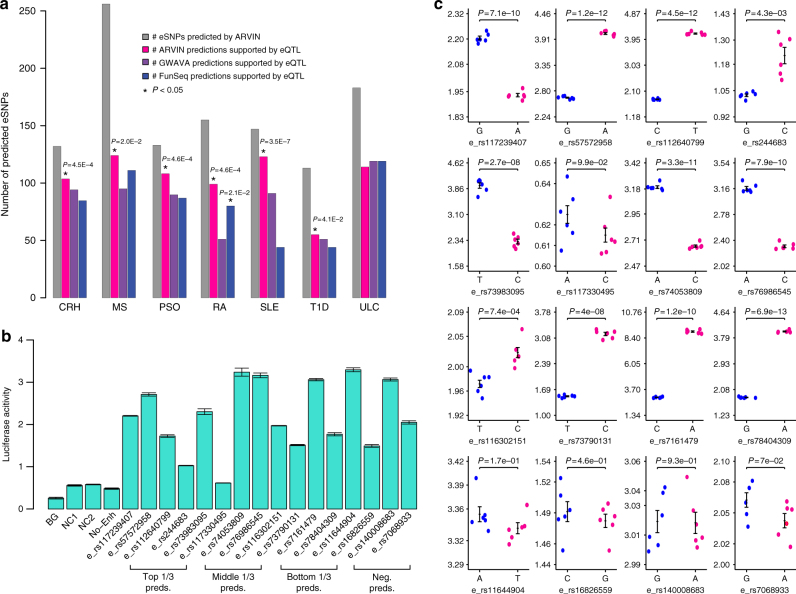
Predicted risk enhancer SNPs in seven autoimmune diseases. **a** Number of predicted risk eSNPs in each disease and overlap with eQTL data. For comparison purpose, the prediction thresholds of GWAVA and FunSeq2 were set to give the same number of predictions as ARVIN. Statistical significance of overlap between predicted eSNPs by a given method and eQTL data were computed using the hypergeometric distribution. *P value < 0.05. CRH, Crohn’s disease; MS, multiple sclerosis; PSO, psoriasis; RA, rheumatoid arthritis; SLE, systemic lupus erythematosus; T1D, type 1 diabetes; ULC, ulcerative colitis. **b** Luciferase reporter assay of enhancers containing predicted risk eSNPs and negative control eSNPs. Reporter activity is relative to co-transfected *Renilla* control. BG, no DNA; NC1 & NC2, negative controls, genomic region without H3K4me1 and H3K27ac signals; No-Enh, construct containing only heat-shock (HS) promoter but no enhancer sequence; Top 1/3 pred., eSNPs in the top 1/3 of predictions by ARVIN, etc. Neg. pred., negative predictions by ARVIN. Values shown are means ± s.e.m. of six replicates. **c** Luciferase reporter activity for both alleles of 12 predicted risk eSNPs (top three rows) and 4 negative control (bottom row) eSNPs. Values shown are means ± s.e.m. of six replicates. *P *values are calculated using two-tailed *t *test

Using ARVIN cutoff that yields an optimal set of predictions (Supplementary Methods, Supplementary Fig. 5), on average, we predicted 160 risk eSNPs for each autoimmune disease (Fig. 4a). We evaluated the predictions using eQTLs identified in disease-relevant tissues by the GTEx consortium and by Westra et al.^30,31^ (Supplementary Table 6). For six out of seven autoimmune diseases, the set of risk eSNPs predicted by ARVIN has significant overlap with eQTLs identified in relevant tissues. In contrast, only predictions by FunSeq2 in one disease (rheumatoid arthritis) have significant overlap with eQTL data (Fig. 4a).

To experimentally test the predicted risk eSNPs, we randomly selected four predicted risk eSNPs with ARVIN scores in the top, middle, and bottom thirds of the score distribution, respectively. As a comparison, we also randomly chose four eSNPs that are negative predictions by ARVIN (Supplementary Table 7). We first used dual luciferase reporter assay to test the activity of the enhancers in CD4^+^ T cells. All 16 enhancers (12 containing predicted risk eSNPs and 4 containing negative predictions) significantly enhance luciferase activity in comparison to the two negative control sequences (Fig. 4b). Next, we compared the enhancer constructs that contain alternative alleles of the predicted eSNPs (Supplementary Table 8). Among the 12 predicted risk eSNPs, 11 show differential enhancer activities (*P* < 0.05) with different alleles of the SNPs. In contrast, none of the negative predictions show significant activity difference between the two alleles of the SNP (Fig. 4c).

### Many genes are targeted by multiple risk noncoding variants

**Fig. 5:**
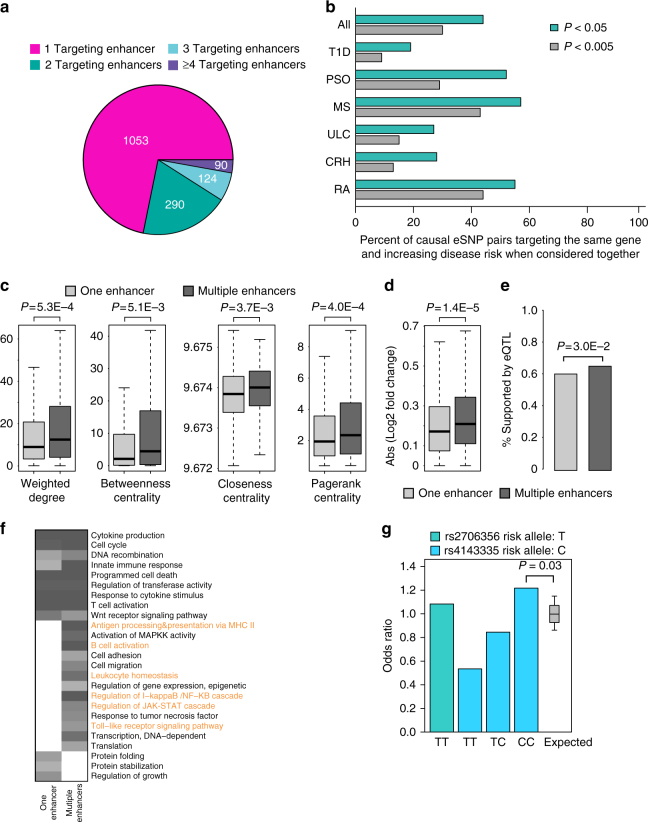
Combinatorial risk noncoding mutations. **a** Number of genes that are targeted by different numbers of eSNP-containing enhancers. A considerable fraction of genes are targeted by multiple enhancers, suggesting combinatorial regulation of affected genes by multiple risk eSNPs. **b** Percentage of causal eSNP pairs that target the same gene and increase disease risk when considered together. Genes targeted by multiple risk eSNPs have higher values of network topological features (**c**), higher expression fold changes between case and control samples (**d**), higher overlap with eQTLs (**e**), and more enriched GO terms for immune responses (highlighted in orange) (**f**). **g** Odds ratio for all individuals homozygous at the eSNP r2706356 and odds ratios determined when homozygous individuals were stratified based on the genotype of the co-targeting eSNP (rs4143335), as compared to the expected distribution of odds ratios

Increasing evidence suggests that many genes are regulated by multiple enhancers during normal and disease development^27,32–35^. This phenomenon suggests that mutations in multiple enhancers of the same gene could collectively contribute to the deregulation of the gene during pathogenesis. Consistent with this hypothesis, among the seven autoimmune diseases, we found that 32% of genes are affected by multiple predicted eSNPs that are located in multiple enhancers targeting these genes (Fig. 5a).

We tested whether two risk eSNPs that target the same gene increase disease risk compared to each eSNP alone. We used GWAS data generated by the Wellcome Trust Case Control Consortium^36,37^ for six autoimmune diseases, including Crohn’s disease, multiple sclerosis, psoriasis, rheumatoid arthritis, type 1 diabetes, and ulcerative colitis.

For all risk eSNP pairs targeting the same gene, we assessed their combined effect on disease risk using a permutation-based procedure^38^ (see Methods). At *P* < 0.05, we found that the percentage of eSNP pairs with increased risk ranges from 19% for type 1 diabetes to 57% for multiple sclerosis with an overall percentage of 44% across the six diseases (Fig. 5b).

Besides risk eSNPs, we further investigated the genes targeted by multiple risk eSNPs. We found several unique features about these genes. First, they tend to have higher network centrality measures (Fig. 5c). Second, their expression levels are more perturbed in disease samples compared to control samples (Fig. 5d). A higher percentage of the regulating risk eSNPs overlap with eQTLs (Fig. 5e). Finally, they are enriched for more Gene Ontology (GO) terms for direct immune responses (Fig. 5f). Taken together, these unique properties of multi-targeted genes suggest they might be rate-limiting genes in disease pathogenesis.

**Fig. 6:**
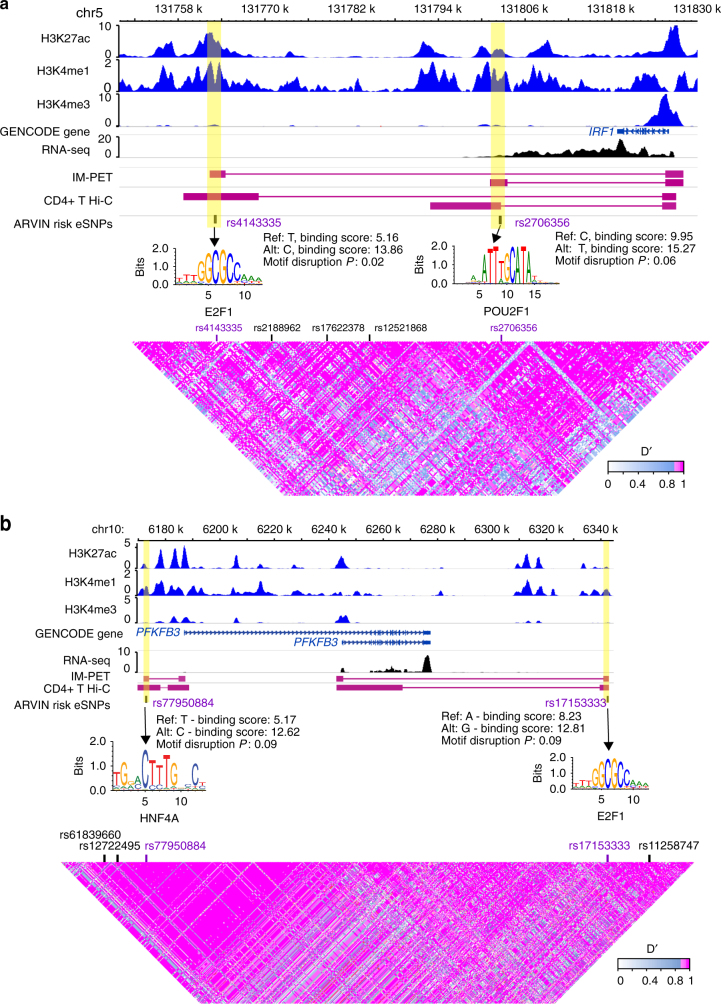
Examples of genes targeted by multiple risk eSNPs. Two genes, *IRF1* (**a**) and *PFKB3* (**b**) targeted by two risk eSNPs. Enhancers are highlighted in yellow shade. IM-PET, enhancer−promoter interactions predicted by IM-PET. CD4^+^ T Hi-C, enhancer−promoter interactions detected by Capture Hi-C data. Annotation for autoimmune disease-associated loci is based on ImmunoBase

Figure 6a, b shows two example genes that are targeted by multiple risk eSNPs. *IRF1* plays a critical role in regulatory T-cell function and autoimmunity^39^. It is targeted by two enhancers based on both IM-PET prediction and experimental Capture-Hi-C data in CD4^+^ T cells^40^. The two eSNPs (rs4143335 and rs2706356) significantly disrupt the binding of HNF4A and E2F1, respectively. Both E2F1^41^ and POU2F1^42^ have been shown to be important transcriptional regulators of CD4^+^ T-cell function. When we determined the clinical risk (odds ratio) for Crohn’s disease based on the genotype of both variants, we found an increase in clinical risk to an odds ratio of 1.22 for individuals homozygous for the risk allele (T) of rs2706356 and homozygous for the C allele of rs4143335 (Fig. 5g, Supplementary Fig. 6). The other example involves the gene *PFKFB3* that encodes a rate-limiting glycolytic enzyme. Deficiency of PFKFB3 has been linked to reprogrammed metabolism in T cells from rheumatoid arthritis patients^43,44^. The two risk eSNPs (rs77950884 and rs17153333) significantly disrupt the binding of HNF4A and E2F1, respectively. Interestingly, in both examples, the lead GWAS SNPs are not predicted to be the risk SNPs, emphasizing the challenge of finding risk SNPs in the presence of genetic linkage.

### Most perturbed subnetwork by all risk eSNPs in a disease

It has been suggested that the effects of multiple low-penetrance enhancer variants can be amplified through coordinated dysregulation of the entire GRN of a key disease gene, as illustrated in an elegant study by Chatterjee and colleagues^35^. To obtain a systems-level view of the pathways collectively perturbed by all risk eSNPs in a disease, we used the Prize Collecting Steiner Tree (PCST) algorithm to identify a connected subnetwork composed of all risk eSNPs and genes bridging the risk eSNPs in the network. By algorithmic design, the resulting subnetwork is maximized for nodes and edges with large weights. In other words, these are downstream genes that have high levels of differential expression and functional interactions. Therefore, the effects of the risk eSNPs are most likely propagated via such a subnetwork.

**Fig. 7:**
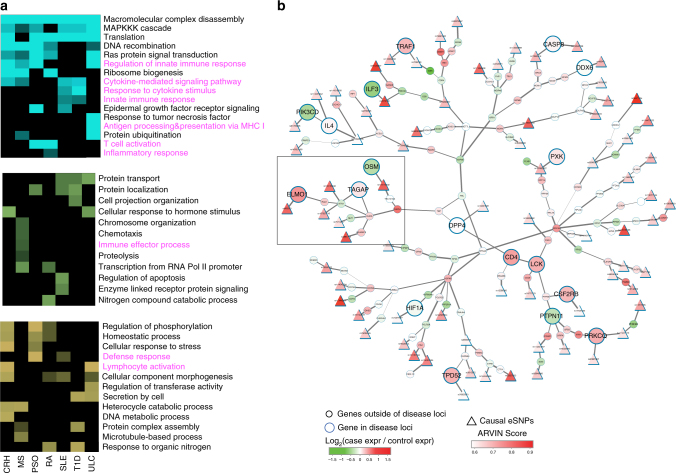
Gene subnetwork collectively perturbed by all risk eSNPs in a disease. **a** Uniquely enriched GO terms among perturbed subnetworks downstream of risk eSNPs predicted by ARVIN (cyan), GWAVA (green), and FunSeq2 (yellow), respectively. GO terms for immune responses are highlighted in magenta. **b** An example of perturbed subnetwork by all risk eSNPs in rheumatoid arthritis. Circle, genes. Node size represents location of a gene relative to disease-associated loci; bigger node, within a disease-associated locus, smaller node, outside a disease-associated locus; node color represents differential gene expression between case and control samples. Triangle, predicted risk eSNPs

For each disease, we compared the subnetworks downstream of risk eSNPs predicted by ARVIN, GWAVA, and FunSeq2, respectively. We found that subnetworks downstream of ARVIN-predicted eSNPs have more enriched GO terms related to immune cell functions (Fig. 7a), further suggesting the predicted upstream eSNPs are more likely to be causal eSNPs.

Figure 7b shows an example subnetwork for rheumatoid arthritis. Such a network view reveals two interesting features of the perturbations caused by risk eSNPs. First, we found that multiple members of a pathway can be targeted by different risk eSNPs. For instance, the subnetwork contains ten genes that are involved in the *RhoA*-mediated small GTPase signaling (highlighted in a square). Six of the ten genes are individually targeted by different risk eSNPs. Rho kinase signaling has been shown to have a critical role in the synovial inflammation of rheumatoid arthritis^45,46^. Second, we found that many genes targeted by risk eSNPs are not located in disease-associated loci. This is consistent with the notion of long-range interaction between enhancers and their target genes. Most perturbed subnetworks for other diseases in this study are shown in Supplementary Fig. 7.

## Discussion

A number of methods have been developed for inferring noncoding risk variants. Although they differ by the computational methodology used, conceptually, all existing methods use sequence and chromatin features around a candidate variant to make a prediction. Transcription regulation occurs in a complex network of regulatory interactions between transcription factors and target genes. To better understand noncoding risk mutations, they should be examined in the context of the regulatory network of disease-relevant cell type(s). To our knowledge, ARVIN is the first method that explicitly uses disease-relevant GRN for finding noncoding risk variants. Disease-specific transcriptomic and epigenomic data are integrated with a probabilistic functional gene interaction network to generate a weighted GRN, which serves to provide disease-specific information and reduce noise at the same time. Using gold-standard noncoding variants, we demonstrate that genes targeted by causal SNPs exhibit characteristic network features compared to genes targeted by non-causal SNPs. The network-based features are complementary to sequence-based features. Combination of both types of features achieves the highest accuracy in predicting causal noncoding mutations. In support of the utility of disease-specific network for finding noncoding risk variants, we found that both the separation of feature values and classification accuracy decrease when non-disease-specific networks are used in ARVIN (Supplementary Fig. 1). Although we focused on common germline variants in this study, ARVIN is also applicable to somatic and rare variants because the same mechanisms of transcriptional regulation are affected by the different types of mutations.

A recent study demonstrated that multiple low-penetrance enhancer variants can cause significant dysregulation of the entire GRN by targeting a key disease gene^35^. Along this line, our systematic analysis of seven autoimmune diseases revealed the abundance of combinatorial risk variants that affect the same gene. This result is supported by the observation that promoters of many genes are physically contacted by multiple enhancers^33,34,47^. Our result suggests that genes affected by combinatorial risk variants tend to be more centrally located in the GRN, have higher expression change in response to disease, and directly mediate immune responses. Taken together, these unique features strongly suggest that genes affected by multiple risk eSNPs may play a rate-limiting role in disease pathogenesis.

Beyond studying individual risk eSNPs, it would be tremendously useful to have a holistic view of the subnetwork jointly perturbed by all risk eSNPs in a disease. To this end, we used the PCST algorithm to identify the core subnetwork that is most perturbed by all risk eSNPs in a disease. Knowledge about the perturbed subnetwork can be used to prioritize genes and variants for follow-up studies. Furthermore, comparative analysis of the perturbed subnetworks in different diseases may lead to novel insights into disease pathogenesis and suggest novel therapeutic strategies.

ARVIN can be improved in a few ways. First, the performance of ARVIN can be affected by the quality of GRN. In this study, we addressed this issue by weighting the edges and nodes in the network. To further examine the robustness of our method, we substituted HumanNet with the functional gene interaction network annotated in the STRING database^48^. Using the same set of gold-standard promoter and enhancer SNPs, we found that ARVIN achieves similar performance gain compared to GWAVA and FunSeq2 (Supplementary Fig. 8). To further evaluate the general applicability of ARVIN on enhancer−promoter networks, we compared the performance of the three methods using alternative tissue-specific networks constructed using enhancer−promoter interactions generated by the FANTOM5 consortium^49,50^. Again we found that ARVIN achieves the best performance (Supplementary Fig. 9).

As more experimental data on molecular interactions become available, they can be used to construct more accurate GRNs. In addition, since ARVIN is a supervised method, its accuracy depends on the training set. The training set we used (HGMD^28^) is the most comprehensive manually curated disease mutation database. It only includes causal diseases variants, excluding those that are associated with the disease due to linkage with another known risk variant^51^. However, it may be possible that some false-positive variants are included due to linkage with yet-to-be discovered causal SNPs. As the annotation for causal variants continue to improve, they can be used to train a more robust classifier.

## Methods

### ARVIN framework

Key components of the computational framework are described in the following sections: construction of disease-relevant GRN, computation of network-based features associated with candidate eSNPs, and classifier for risk eSNPs using genomic, epigenomic, and network-based features.

### Construction of disease-relevant gene regulatory network

Network construction starts with identifying eSNPs. For each lead GWAS SNP, we identify the LD block to which it belongs. We then intersect the set of SNPs in the LD block with the set of enhancers from cell/tissue types relevant to the disease. This gives us a set of enhancer SNPs (eSNPs) in a given LD block identified by the lead GWAS SNP.

The GRN consists of two types of nodes, representing eSNPs and genes, and two types of edges, those between eSNPs and gene(s) (denoted as EP edges) and those between genes (denoted as FI edges) (Fig. 1c). EP edges represent regulatory relationship between an enhancer and its target(s). FI edges represent functional interactions between genes. EP edges are based on enhancer−promoter interactions predicted by the IM-PET algorithm^27^ (Fig. 1a). Note that the enhancer−promoter interactions are also predicted using ChIP-Seq and gene expression data from cell/tissue types relevant to the disease. FI edges are taken from HumanNet, which is a probabilistic functional gene network of 16,222 protein-encoding genes in humans^21^. Each interaction in HumanNet has an associated probability representing a true functional linkage between two genes. It is constructed by a Bayesian integration of 21 types of “omics” data including physical interactions, genetic interactions, gene co-expression, literature evidence, homologous interactions in other species, etc. HumanNet has been successfully used for improving inference accuracy of coding variants. Interactions in HumanNet are not disease-specific, to add disease-specific information for the functional gene interaction network, we add differential gene expression information from case vs control comparison in disease-relevant cells/tissues.

Nodes and edges in the network were weighted to (1) take into account the noise in the data; (2) to represent the relative importance of different genes and interactions. Weights for eSNPs, *W*^eSNP^, are based on the *P *value of disrupting putative transcription factor binding site due to the SNP. Weights for genes, *W*^DE^, are based on the *P *values of differential gene expression between case and control samples. Weights for EP edges, *W*^EP^, are based on the probability for enhancer−promoter interaction outputted by the IM-PET algorithm. Weights for FI edges, *W*^FI^, are taken from HumanNet. To make the values of each type of weights comparable, we performed min-max normalization for each type of weights.

### Network-based features associated with candidate eSNPs

We compute five network-based features. The first one is module score, which is based on the gene modules downstream of an eSNP. Our overall hypothesis is that a causal eSNP contributes to disease risk by directly causing expression changes in genes of disease-relevant pathways. Thus, in addition to the direct target gene of the eSNP, other genes in the same pathway can also provide discriminative information. With the weighted GRN, our goal is to identify “heavy” gene modules in the network that connects a given eSNP to a set of genes (encircled modules in Fig. 1c), hereby termed eSNP module. On the other hand, non-causal eSNPs are expected to be associated with “light” modules, i.e. having marginal impact on pathway gene expression (e.g. eSNP3 in Fig. 1c). To score a candidate module, we use the following additive scoring scheme by summing up all node and edge weights divided by the number of nodes (*N*) in the candidate module.

$${{S}}\, =\, ({{W}}^{{\rm {eSNP}}}\, +\, {{W}}^{{\rm {DE}}}\, +\, {{W}}^{{\rm{EP}}}\, +\, {{W}}^{{\rm{FI}}})/{{N}}.$$

We conduct module search from all eSNPs in the weighted network. It is an NP-hard problem to obtain a global optimal solution consisting of all heavy subnetworks. We thus use a greedy search strategy. Starting with each eSNP, our algorithm considers all genes connected to the current eSNP-module and add the node whose addition leads to the maximal increase of the scoring function. This procedure repeats until there is no node whose addition can improve the module score. Several recent studies have reported that multiple enhancer elements could be present at a single GWAS locus^52,53^. Our network-based framework can naturally handle such cases because we consider all eSNPs simultaneously during module search. We assessed the statistical significance of candidate modules using randomized networks. Specifically, for edges, we randomized them by edge-preserved shuffling. For nodes, we randomly shuffled their values within each type (i.e. among genes or among eSNPs). The empirical *P*values are computed based on the null score distribution from the randomized networks.

The second network-based feature is weighted degree of a node *v* directly downstream of an eSNP. It is defined as ∑_(*u*,__*v*)__∈*E*_*W*(*u*,*v*), (where *W*(*u*,*v*) is the edge weight for the edge connecting nodes *u* and *v*.

The third network-based feature is betweenness centrality of a node *v* directly downstream of an eSNP. Betweenness centrality of a node in a network corresponds to the proportion of shortest paths in the network going through node. The raw betweenness centrality is defined as *C*_B_(*v*) = ∑_*v*__≠__*t*__≠__*u*_ (*σ*_*tu*_ (*v*))/*σ*_*tu*_, where *σ*_*tu*_ is the total number of shortest paths between node *t* and node *u*. *σ*_*tu*_(*v*) is the subset of *σ*_*tu*_ that go through *v*. The normalized betweenness centrality is defined as $C{\prime}_{\rm{B}}\left( {{v}} \right)\, =\, {{C}}_{\rm{B}}\left( {{v}} \right) \,\times\, {{N}}$, where *N* is the total number of nodes in the network.

The fourth network-based feature is closeness centrality of a node *v* directly downstream of an eSNP. Closeness centrality is the inverse of the sum of shortest paths between a node and all nodes in a network. It is proportional to the time by which information spreads from the node of interest to all other nodes in the network. The raw closeness centrality is ${{C}}_{\rm{C}}({{v}})\, = \,{1}/{{\mathop {\sum }\nolimits_{{{u}} \ne {{v}}} {{d}}\left( {{{u}},{{v}}} \right)}}$, where *d*(*u*,*v*) indicates the length of the shortest path between *u* and *v*. The normalized closeness centrality is defined as ${{C{\prime}}}_{\rm{C}}({{v}})\, = \,{{C}}_{\rm{C}}({{v}})\,\times\,{{N}}$, where *N* is the total number of nodes in the network.

The fifth network-based feature is page rank centrality of a node *v*directly downstream of an eSNP. Page rank centrality is a network measure based on the idea that the importance of a given node is determined by itself and its neighbors’ importance. Page rank centrality of a node vis defined as *C*_P_ (*v*) = (1−*d*)/(*N*)+∑_*v*∈*V*(*v*)_ (*C*_P_ (*v*))/*L*(*v*), where *V*(*v*) is first neighbors of node *v* and *L*(*v*) is the set of edges incident on node *v*. *d* denotes a damping factor adjusting the derived value downward and *N* is the total number of nodes in the network. The normalized page rank centrality is defined as ${{C{\prime}}}_{\rm{P}}({{v}})\, = \,{{C}}_{\rm{P}}({{v}})\,\times\,{{N}}$.

### Predicting risk variants

To classify risk eSNPs, we trained an RF classifier using the combined feature set that consists of 5 network-based features, 6 binary features from FunSeq, and 175 features from GWAVA. The classifier contained 500 decision trees. Each decision tree was built using ~20% of randomly selected training data (100 out of 464) and $\sqrt {187}\, \approx\, 14$ randomly selected features. Classification error was measured with data not used for training (i.e. out of bag data). To compute feature importance, for each decision tree, the classification error was computed using permuted and non-permuted feature values. The difference between the two classification errors were then averaged over all trees and used as feature importance.

To select most predictive features, we used an RFE strategy^54^. At each iteration of the feature selection, the top *S* most important features were selected. The RF model was refit and corresponding performance was evaluated. To access the variance in performance at each iteration of feature selection, we did fivefold cross-validation. After all iterations, the optimal set of features was determined using the subset with best average performance across fivefold cross-validation. Receiver operating characteristic (ROC) curve is used to evaluate prediction performance. Difference in auROC between two ROC curves is computed using a bootstrap-based method^55^.

Based on the optimal set of features, we build an RF classifier. Given a genetic variant along with its feature values, the classifier outputs a prediction probability indicating how likely this genetic variant is a risk variant in a given disease.

### Predictions of enhancers and enhancer−promoter interactions

Enhancers were predicted using the Chromatin Signature Inference by Artificial Neural Network CSI-ANN algorithm^10^. The input to the algorithm is the normalized ChIP-Seq signals of three histone marks (H3K4me1, H3K4me3, and H3K27ac). The algorithm combines signals of all histone marks and uses an artificial neural network-based classifier to make predictions of active enhancers with the histone modification signature “H3K4me1^hi^ + H3K4me3^neg/lo^ + H3K27ac^hi^”. The training set for the classifier was prepared using ENCODE data of mouse ES-Bruce4, MEL, and CH12 cell lines. To create the training set for active enhancers, we first selected a set of promoter-distal p300 binding sites (2.5 kb from Refseq TSS), and overlapped them with the histone modification peaks. The top 300 distal p300 sites that overlapped with H3K4me1 and H3K27ac peaks, but not H3K4me3 peaks, were selected as the positive set. One thousand randomly selected genomic regions and 500 active promoter regions were used as the negative set. Enhancers were predicted using a false discovery rate (FDR) cutoff of 0.05. Predicted enhancers that overlapped by at least 500 bp were merged by selecting the enhancer with the highest CSI-ANN score. We obtained histone modification ChIP-Seq data from the NCBI Epigenome Atlas, Roadmap Epigenomics Project, Encyclopedia of DNA Elements (ENCODE), International Human Epigenome Consortium, and the GEO database (Supplementary Table 1).

Target promoter(s) of an enhancer were predicted using the IM-PET^27^ algorithm. It predicts enhancer−promoter interactions by integrating four features derived from transcriptome, epigenome, and genome sequence data, including: (1) enhancer−promoter activity correlation, (2) transcription factor-promoter co-expression, (3) enhancer−promoter co-evolution, and (4) enhancer−promoter distance. Here, we used tissue/cell type-specific histone modification ChIP-Seq and RNA-Seq data (Supplementary Table 1) to compute values of features 1, 3, and 4 for the given tissue/cell type. Values of feature 3 were based on sequence conservation across 15 mammalian species (human, chimp, gorilla, orangutan, gibbon, rhesus, baboon, marmoset, tarsier, mouse lemur, tree shrew, mouse, rat, rabbit, and guinea pig). We used an FDR cutoff of 0.05 as the threshold for making predictions.

### Evaluation of enhancer−promoter predictions

We searched for large-scale chromatin interaction data measured using either Hi-C or ChIA-PET protocol (Supplementary Table 2). We used the reported EP interactions in these studies as the gold standard to assess the quality of our predicted enhancer−promoter pairs. We first identified EP pairs in which the enhancers overlap with the interacting fragments reported by Hi-C or ChIA-PET studies. Those EP pairs are regarded as eligible for comparison with Hi-C or ChIA-PET data. We then computed the ROC curves using EP interactions reported in either Hi-C or ChIA-PET studies as the gold standard.

### Gold-standard risk variants located in gene promoters

The Human Gene Mutation Database (HGMD, version 2014 r1)^28^ was used to select regulatory variants located in promoter region that was defined as 2 kb upstream and 0.5 kb downstream of TSS. Transcript annotation was based on Gencode v19 (GRCh37). Only transcripts with high confidence were used (level <3). We selected all diseases and their associated SNPs in HGMD that satisfied the following three criteria: (1) SNPs have the annotation of “DP” (disease-associated polymorphism), or “FP” (polymorphism exerts a direct functional effect), or “DFP” (disease-associated polymorphism with additional supporting functional evidence) or “DM” (disease causing mutation) in HGMD; (2) case and control gene expression data were available for the disease; (3) genes of the reported promoter were present in the HumanNet connected network. For negative control SNPs, we used common (minor allele frequency ≥ 1%) SNPs from the 1000 Genomes Project. Seventy-five percent of the HGMD variants lie within a 2 kb window flanking the transcription start site^16^. Therefore, we selected negative control SNPs such that the distance distribution to the nearest TSS matches that of the positive training set in order to control for the bias in the positive set. The lists of positive and negative control variants are provided in Supplementary Data 1.

### Processing of gene expression profiling data

All gene expression microarray data were analyzed using the limma package^56^. Raw microarray data were background corrected and quantile normalized. Linear model was fit to the data using the lmFit function of limma. Differential expression was assessed at probe level using the empirical Bayes (eBayes) method. To summarize differential expression at gene level, we selected the minimum *P* value across the probes that match to a gene. The list of gene expression data sets used in this study to assess differential expression is provided in Supplementary Table 3.

### Gold-standard risk variants located in enhancers

We curated a set of experimentally validated eSNPs from multiple resources, including HGMD^28^, ClinVar^57^, Open Regulatory Annotation Database (OregAnno) ^58^, and manual search of PubMed literature. We accepted an eSNP as being validated only if it satisfies the following criteria: (1) significant association of the eSNP with the disease; (2) there is direct experimental evidence that the GWAS SNP causes differential TF binding and gene expression change; and (3) the enhancer is located more than 5 Kbp away from the affected gene promoter. The list of experimentally validated eSNPs is provided in Supplementary Table 4.

### Identification of linkage equilibrium blocks

We used data from the 1000 Genomes project (phase 3 release) to identify SNPs in the same LD with experimentally validated enhancer SNPs and GWAS catalog lead SNPs. PLINK^59^ was used to identify linked SNPs with D′ > 0.9 and within 1 Mb from either validated enhancer SNPs or GWAS lead SNPs. SNPs with D′ > 0.9 with the index SNP are considered in the same LD block as the index SNP.

### FunSeq2 and GWAVA features

FunSeq2^20^ employs seven binary and four continuous features to determine if a variant is deleterious, including: (1) overlap with ENCODE annotation of *cis*-regulatory elements such as enhancer, promoter, or DHS; (2) overlap with sensitive region (i.e. high level of negative selection); (3) overlap with ultrasensitive region; (4) overlap with ultra-conserved elements; (5) overlap with HOT (highly occupied by transcription factors); (6) overlap with regulatory elements associated with genes; (7) recurrence in multiple samples; (8) Motif-breaking score; (9) Motif-gaining score; (10) Network centrality score; and (11) GERP score. Feature values for candidate SNPs were obtained by SNP coordinates to FunSeq2 web portal.

GWAVA uses^16^ 175 genomic and epigenomic features including overlap with histone modification and Transcription Factor ChIP-Seq peaks. We obtained GWAVA feature values for candidate SNPs using the various annotation data sources and Python script (gwava_annotate.py) provided in the GWAVA supplementary portal.

### Identifying the subnetwork affected by a set of risk eSNPs

To identify the subnetwork collectively affected by a set of risk eSNPs in a disease, we use the PCST algorithm. Given an undirected graph *G=*(*V*,*E*,*c,p*), where vertices *V* are associated with non-negative profits *p* and edges *E* are associated with non-negative costs *c*. The PCST algorithm finds a connected subgraph *G′*= (*V′*, *E′*) of *G* that maximizes the net profit which is defined as the sum of all node-associated profits minus all edge-associated costs^60^. The algorithm takes as the input the disease-relevant regulatory network and all risk eSNPs implicated in a given disease. Every input eSNP is considered as a possible root node of the Steiner tree but the one resulting in a Steiner tree with the largest profit is chosen as the final root node. To identify the optimal solution, the algorithm will link every input eSNP to the selected root node maximizing the net profit. This can be solved using message-passing technique^61^. We convert our edge score into edge cost by 1−*S* (*i*,*j*), where *S* (*i*,*j*) is the edge score. The final output of the algorithm is a tree composed of all risk eSNPs and genes that are targeted by them. The eSNPs are connected via interactions among the target genes.

### Generation of non-disease-specific networks

For studying risk variants in promoter, we used the following procedure to construct non-specific networks: (1) using only backbone HumanNet without adding disease-specific differential gene expression information (resulting network termed “No-DE” network); (2) using backbone HumanNet and add differential expression information averaged over all diseases in this study (resulting network termed “AVG-DE” network; (3) using backbone HumanNet and add differential expression information from mismatched cell/tissue types, e.g. when studying heart disease variants, using intestine gene expression data (resulting network termed “Mismatch-DE” network).

For studying risk variants in enhancers, we used the same procedure to create non-specific gene functional interaction network (i.e. FI edges). In addition, for the EP interaction (EP edges), we similarly removed, averaged, and shuffled EP interaction scores but kept the same topology respectively to make EP interactions non-disease specific.

### *P *value for eSNPs that disrupt transcription factor binding sites

For each eSNP, we first scan sequences containing the eSNP using TF binding motifs from the Cis-BP database^62^ and calculate the log-odds ratio score for the SNP-containing sequence. If at least one allele for the SNP has a score greater than the threshold that corresponds to a *P* value 4×10^−7^, which is computed using TFM-Pvalue method^63^ for each motif separately, the sequence is considered as a TF binding site.

Next, the difference in the motif score between the two alleles is computed and compared to a null distribution of motif score differences using one million randomly selected SNPs reported by the 1000 Genomes project. Raw *P *value is corrected for multiple testing using the Benjamini−Hochberg method. The motif disruption score for a given eSNP is the negative logarithm of the most significant motif disruption *P *value among all TF motifs having a binding site overlapping with the eSNP.

### SNPs associated with autoimmune diseases

We obtained SNPs associated with seven autoimmune diseases from the GWAS Catalog^29^. All SNPs have a genome-wide association *P *value of 5×10^−8^ or less. We identified SNPs in the same LD with the GWAS catalog SNPs. Summary of GWAS Catalog SNPs and linked eSNPs is provided in Supplementary Tables 5.

### Identification of optimal set of risk eSNPs in a disease

ARVIN computes a probability score for each candidate eSNP. In order to choose a cutoff for final predictions, we developed the following procedure based on the assumption that a true risk eSNP should either be a lead or linked to a lead GWAS SNP. We first rank all eSNPs in descending order of their ARVIN scores. Next, we compute a cumulative enrichment score as following:

$${{S}}\,=\,\mathop {\sum }\limits_{{{i}}\,=\,1}^{{n}} \left\{ {\begin{array}{*{20}{c}} {d \times {{p}}_{{i}}} \\ {d \times (1 - {{p}}_{{i}})} \end{array}} \right.$$

where *p*_*i*_ is the ARVIN score for eSNP *i* and *d* is an indicator function whose value depends on whether the SNP is located in a disease-associated region, which is defined as the LD block anchored by a GWAS or ImmunoChIP^64^ lead SNP with an association *P *value < 5×10^−8^. *d* takes the value of 1 if eSNP *i* is in a disease-associated region, otherwise the value is −1. Based on this scoring scheme, eSNPs located outside of disease-associated regions contributes negative value to the enrichment score (Supplementary Figure 2). When *S* reaches the maximum value, we use the index *i* as the optimal number of eSNPs for a given disease.

### Evaluation of disease risk of predicted eSNPs with GWAS data

GWAS data for case and control samples were obtained from the WTCCC (Wellcome Trust Case Control Consortium). Samples with reported poor quality were excluded from the analysis. We used data from WTCCC1^37^ data sets for Crohn’s disease (1738 cases), rheumatoid arthritis (1860 cases), type 1 diabetes (1963 cases), and shared control samples from National Blood Service (NBS) individuals (1456 controls). We used WTCCC2^36^ data sets for multiple sclerosis (9770 cases), psoriasis (2178 cases), ulcerative colitis (2361 cases), and shared control samples from NBS phase-2 individuals (2679 controls). Following the best practice guidelines of IMPUTE2,^65^ we imputed 1000 Genomes Phase 1 variants into each GWAS sample. We made hard genotype calls by applying a threshold of 0.9 to the maximum posterior probability of three possible imputed genotypes.

We assessed the combined effect of predicted risk eSNP pairs targeting the same gene on disease risk using a permutation-based procedure^38^. First, for each eSNP pair, we calculated odds ratios for each genotype involving a single SNP. We then calculated odds ratios for nine genotype combinations involving both eSNPs. Next, for individuals of each genotype of the first eSNP in the pair, we randomly assigned a genotype for the second eSNP while maintaining the minor allele frequency of the second eSNP. We generated 1000 permutations and calculated odds ratios for nine genotype combinations. Finally, to assess the significance of the risk alteration, we calculated empirical *P*values by comparing the odds ratio for real genotype pairs and distribution of odds ratio from randomized genotypes.

### Luciferase reporter assay

Jurkat cells were purchased from ATCC (TIB-152). The cell line was tested for mycoplasma contamination using ABI MycoSEQ mycoplasma detection assay (Applied Biosystems). Enhancer sequences containing predicted risk eSNPs were cloned using In-Fusion HD Cloning Kit (Clontech, Cat # 639648) into a luciferase reporter construct pGL3-HS in which expression of the luciferase gene is driven by a minimal heat-shock promoter. Sanger sequencing was used to determine the alleles of the risk eSNPs. Two control regions of ~2 kb without either H3K4me1 or H3K27ac signals were cloned into the same plasmid as negative controls. Reporter constructs were transfected into Jurkat cells using *TransIT*-Jurkat Reagent (Mirus Bio, MIR 2120). As an internal control, a plasmid containing *Renilla* luciferase (pRL-TK from Promega) was co-transfected at a molar ratio of 1:10 for *Renilla* vs firefly luciferases. Cells were collected 48 h post transfection and luciferase reporter levels were measured and compared to *Renilla* luciferase reporter activity using the Dual-Luciferase Reporter Assay kit (Promega, cat # E1910). Primer sequences for cloning enhancers and mutagenesis are listed in Supplementary Tables 7 and 8.

### Site-directed mutagenesis of enhancer SNPs

For mutating a SNP within the tested enhancers, the Q5 site-directed mutagenesis kit (NEB, cat # E0554S) was used according to vendor’s manual. Briefly, primer pairs containing the desired mutations were used to generate plasmids with mutations using the original plasmids as the templates. Sanger sequencing was performed to confirm mutations.

### Data availability

We have deposited ARVIN code, accessory scripts, data and documentation at GitHub with the following url address: https://github.com/gaolong/arvin.

## Electronic supplementary material

Supplementary Information

Description of Additional Supplementary Files

Supplementary Data 1

Supplementary Data 2
