# Supplementary material for: Identifying noncoding risk variants using disease-relevant gene regulatory networks
Source: Nat Commun. 2018 Feb 16;9:702. doi: 10.1038/s41467-018-03133-y (PMC5816022; doi:10.1038/s41467-018-03133-y)
Supplement: Supplementary file 1 — Supplementary Information [file 41467_2018_3133_MOESM1_ESM.docx]

**Supplementary Figure 1. Evaluation of the utility of disease-specific network in predicting risk noncoding variants.** **(a)** Discriminative power using disease-specific network on promoter variants. **(b)** Discriminative power using non-specific backbone HumanNet on promoter variants (No-DE network). **(c)** Discriminative power using backbone HumanNet plus averaged differential expression information on promoter variants (AVG-DE network). **(d)** Discriminative power using backbone HumanNet plus mismatched differential expression information on promoter variants (Mismatch-DE network). **(e)** Receiver Operating Characteristic (ROC) curves using known risk SNPs located in gene promoters. P-values are for the difference in AUC for methods using disease-specific vs using non-disease-specific networks. **(f)** Performance benchmarking using known risk SNPs located in enhancers.


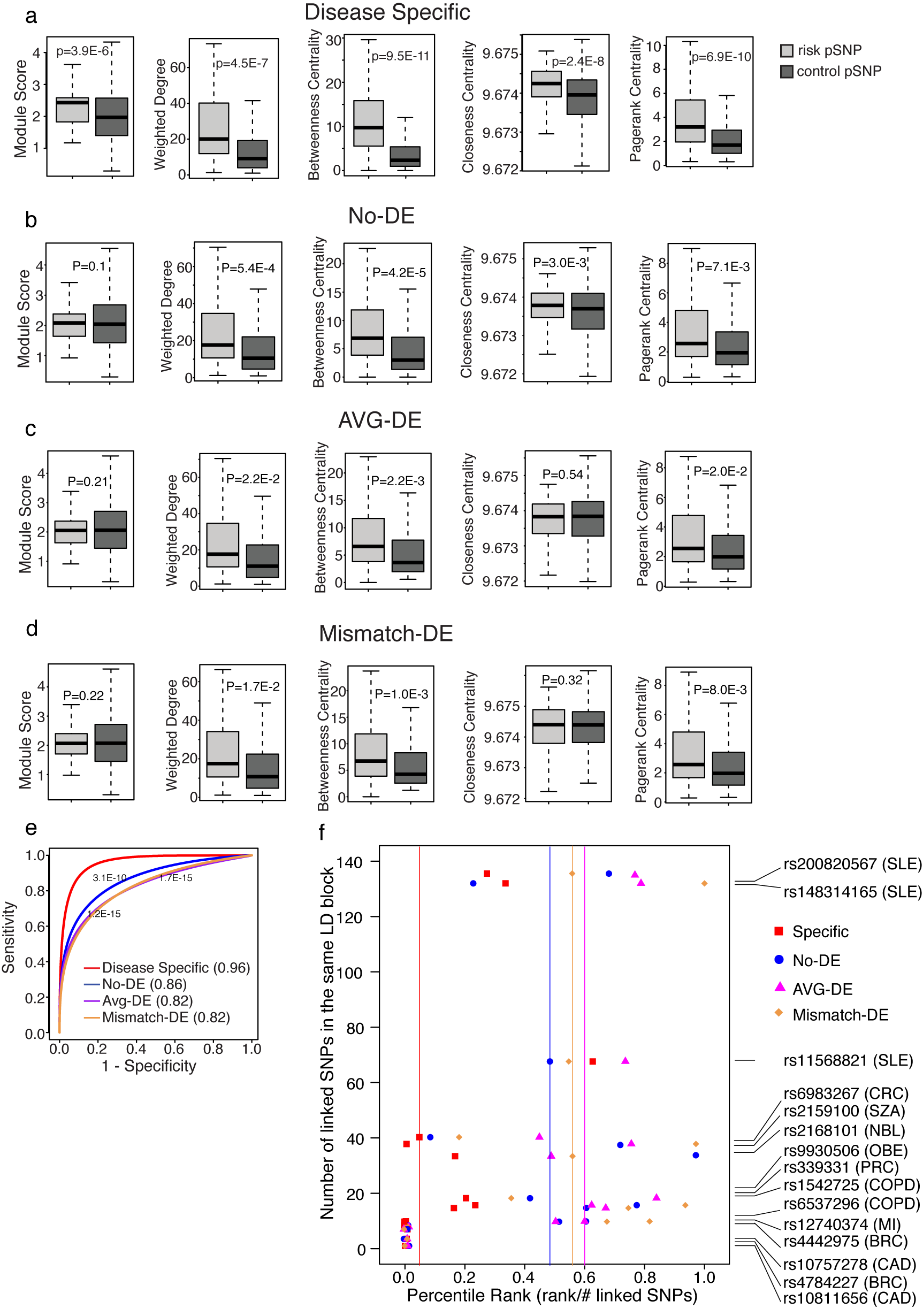


**Supplementary Figure 2. Feature selection using the recursive feature elimination algorithm.**

**
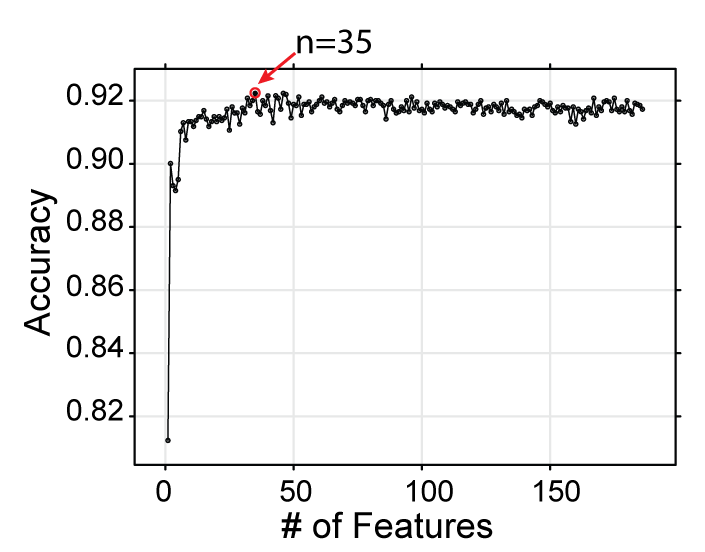
**

**Supplementary Figure 3. Distribution of values of selected GWAVA and FunSeq2 features.** Risk, feature values of risk SNPs. Control, feature values of control SNPs. Bar plot is used for binary features and boxplot is used for real-valued features. P-value is based on t-test.


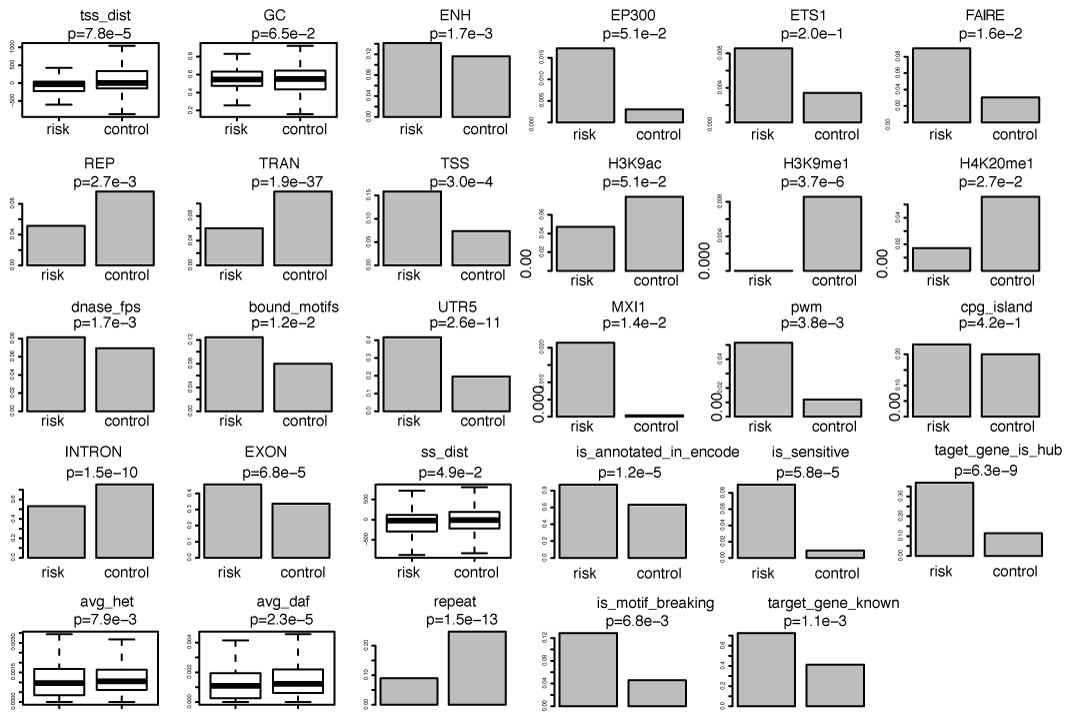


**Supplementary Figure 4. Violin plot of the feature association score between feature pairs.** We computed interaction scores between pairs of sequence-based features and interaction scores between sequence-based features and network-based features. We found that the interaction scores between network-based features and sequence-based features are significantly higher than interaction scores between pairs of sequence-based features. The feature association (interaction) is based on the difference between paired variable importance and additive variable importance. A larger difference indicates higher likelihood of interaction between two variables. NF, network-based feature; SF, sequence-based feature. The violin on the left represents interaction scores between a network feature and a sequence-based feature. The violin on the right represents interaction scores between a pair of sequence-based features.


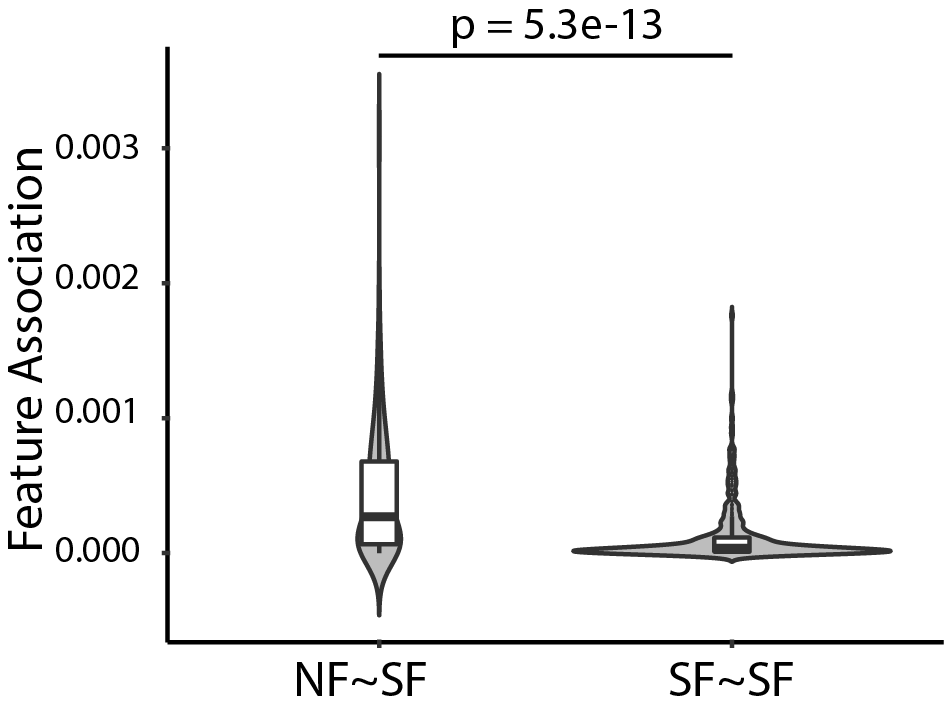


**Supplementary Figure 5. Cumulative enrichment score distribution during the selection of optimal set of risk eSNPs.** CRH, Crohn’s disease; MS, multiple sclerosis; PSO, psoriasis; RA, rheumatoid arthritis; SLE, systemic lupus erythematosus; T1D, type 1 diabetes; ULC ulcerative colitis. Dashed line indicates the maximal value of enrichment score. Values are the number of risk eSNPs selected by the procedure.


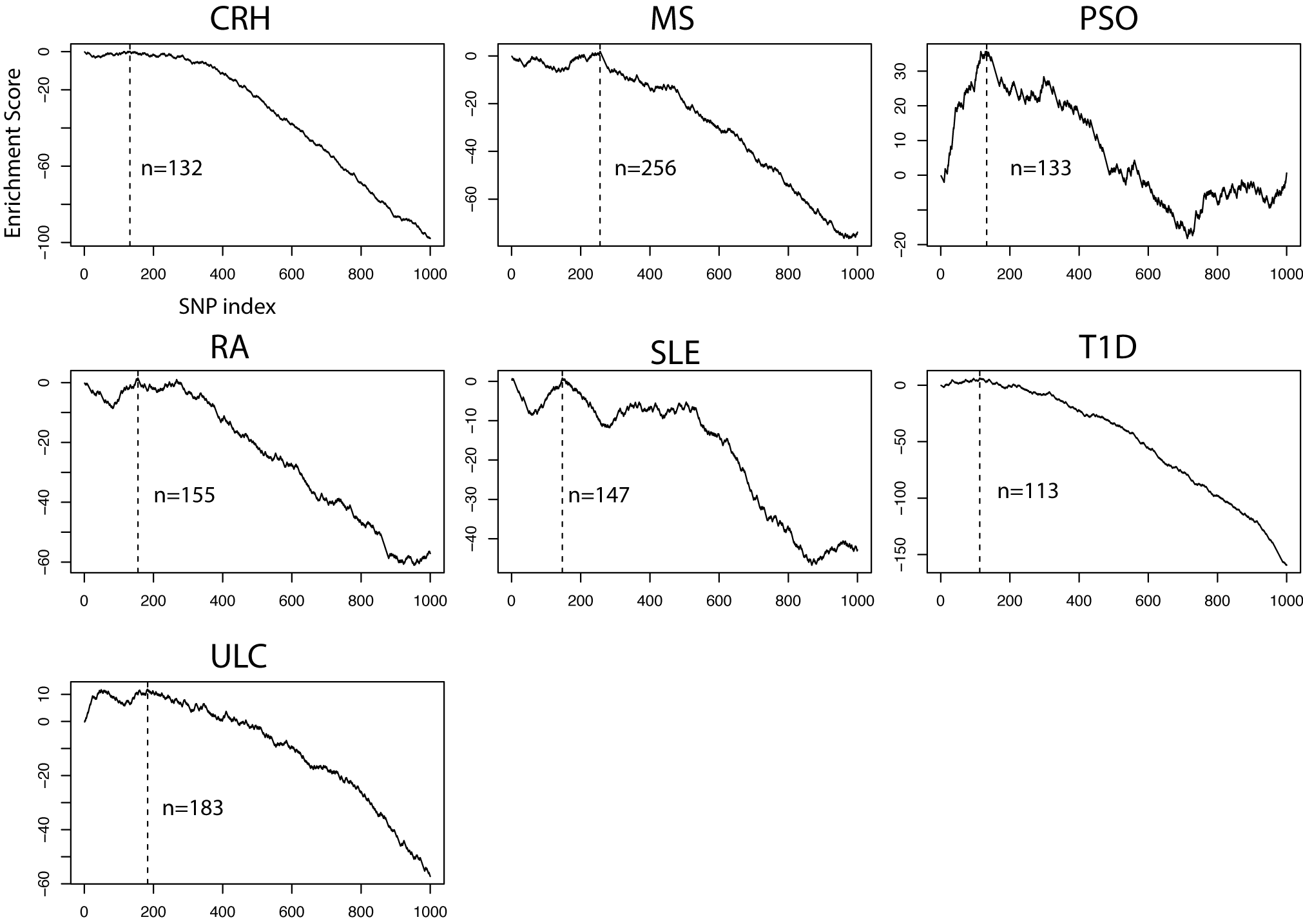


**Supplementary Figure 6. Odds ratios for all genotype combinations of risk eSNP pairs, rs2706356 and rs4143335, for Crohn’s Disease.** Odds ratio for the eSNP r2706356 is shown (left) and combined odds ratios determined when homozygous individuals are stratified based on the genotype of the co-targeting eSNP (rs4143335), as compared to the expected distribution of odds ratios (right).

**
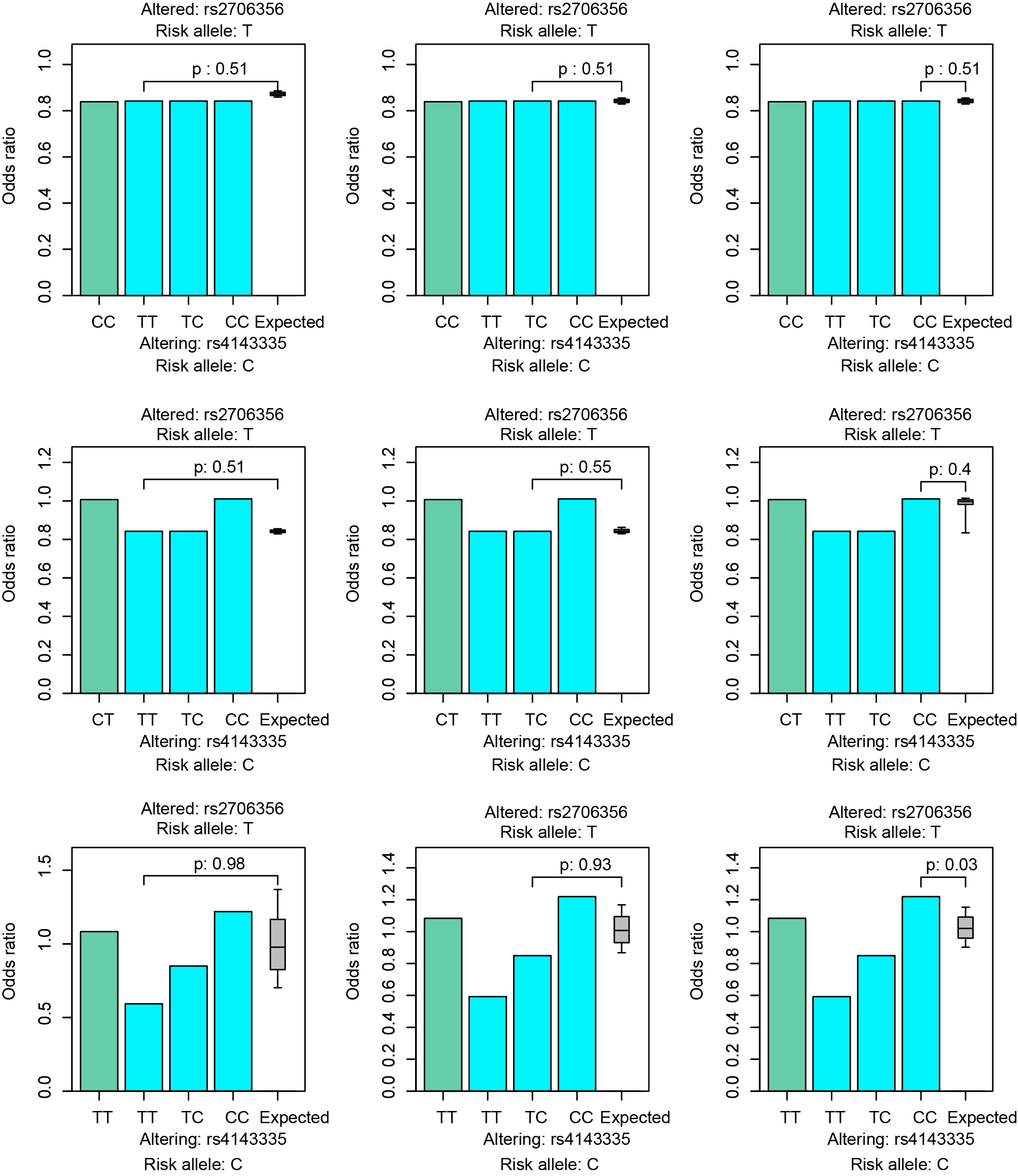
**

**Supplementary Figure 7. Most perturbed subnetwork by all risk eSNPs in a disease.** (**a**) Crohn’s disease. (**b**) multiple sclerosis. (**c**) psoriasis. (**d**) systemic lupus erythematosus. (**e**) type 1 diabetes. (**f**) ulcerative colitis. Circle, genes. Node size represents location of a gene relative to disease-associated loci; bigger node, within a disease-associated locus, smaller node, outside a disease-associated locus; Node color represents differential gene expression between case and control samples. Triangle, predicted risk eSNPs.


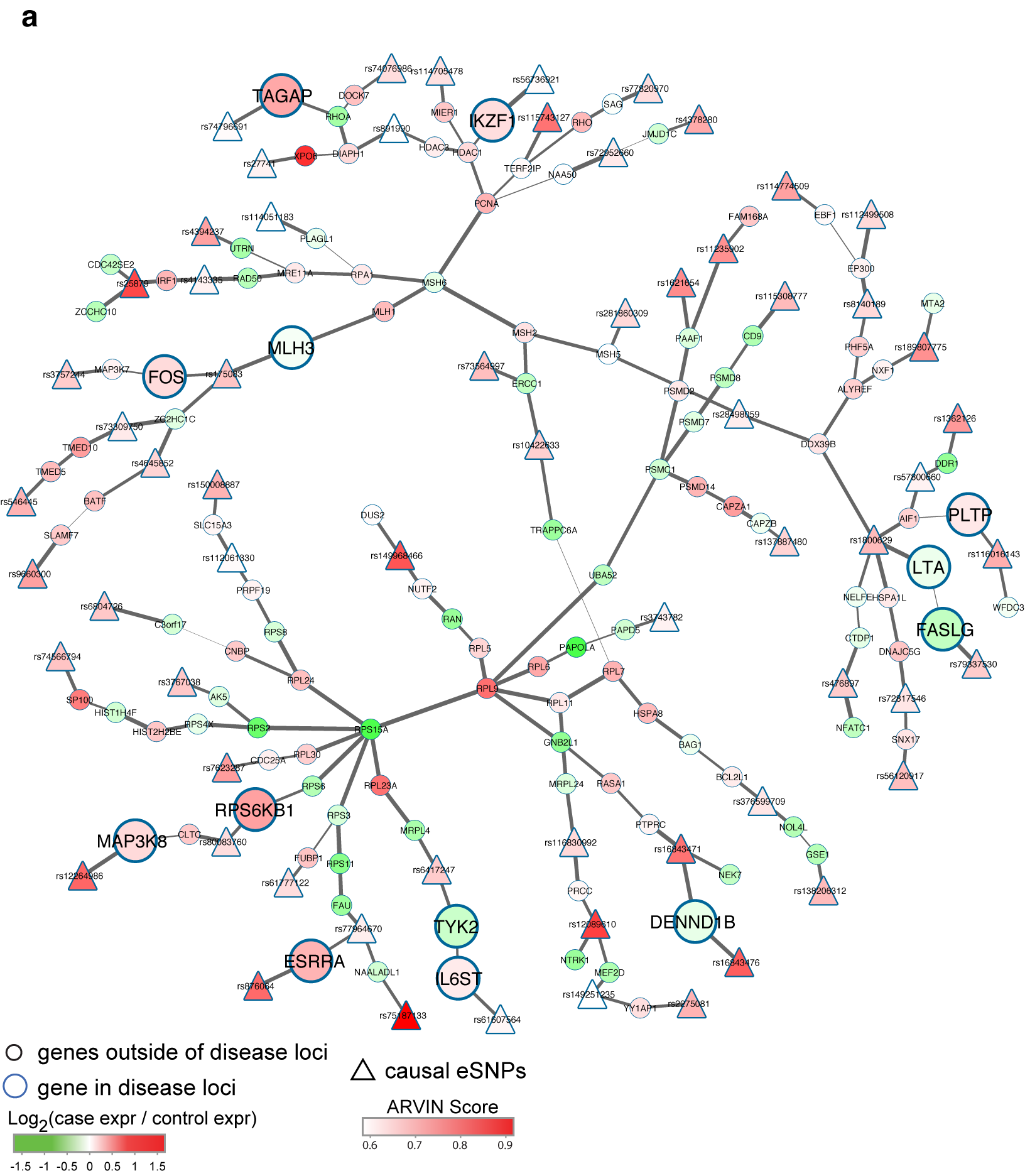


**
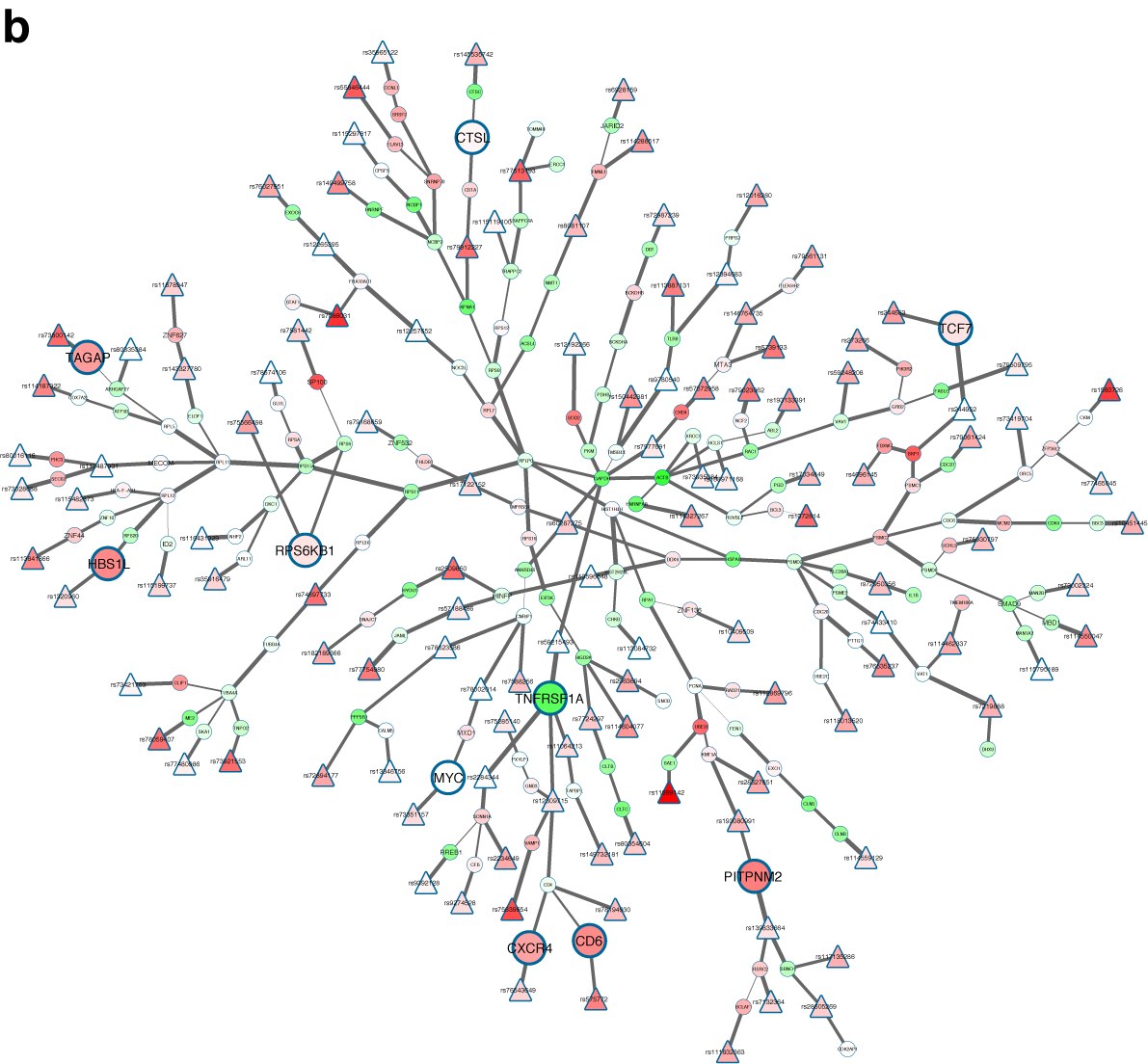
**

**
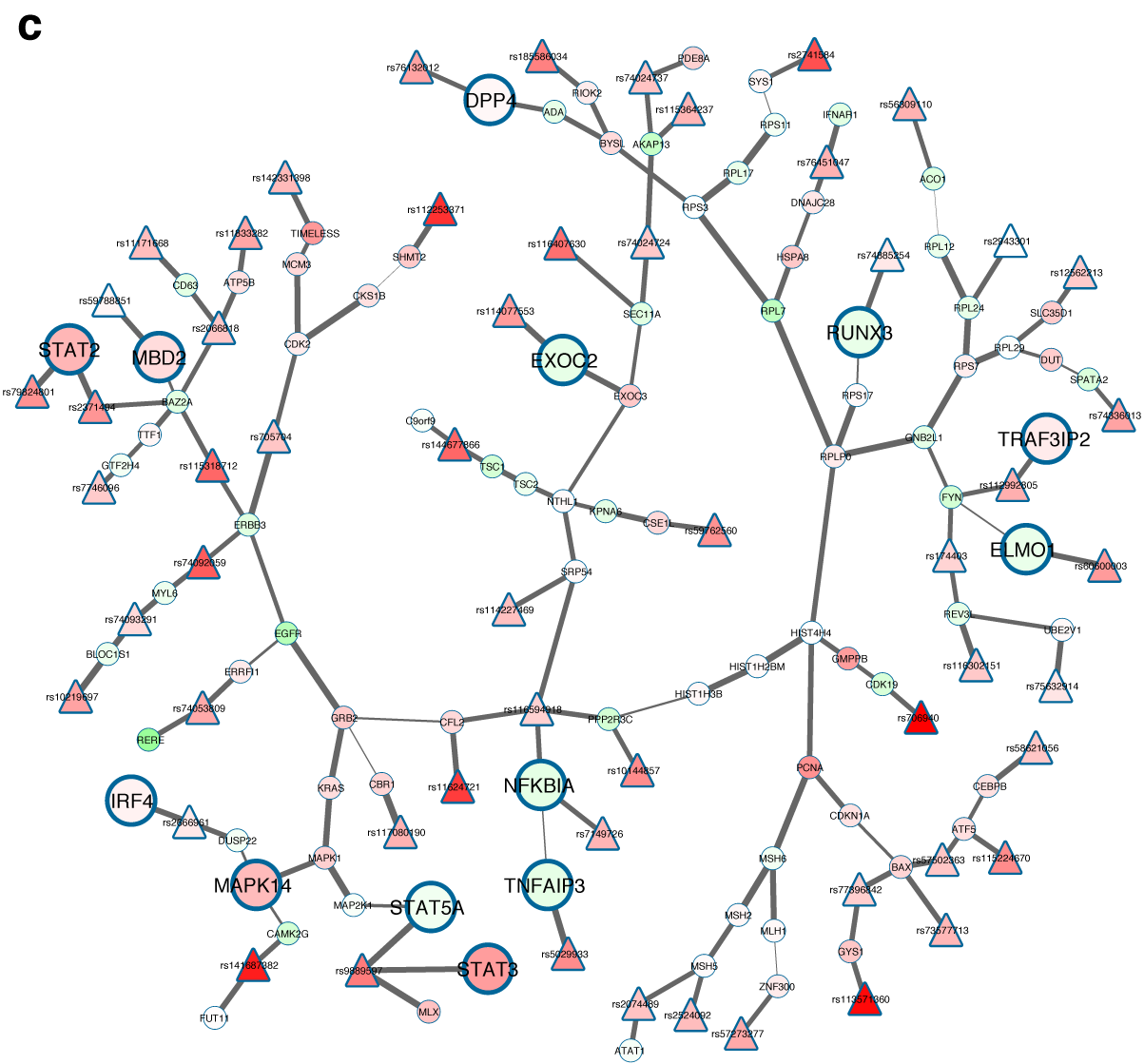
**

**
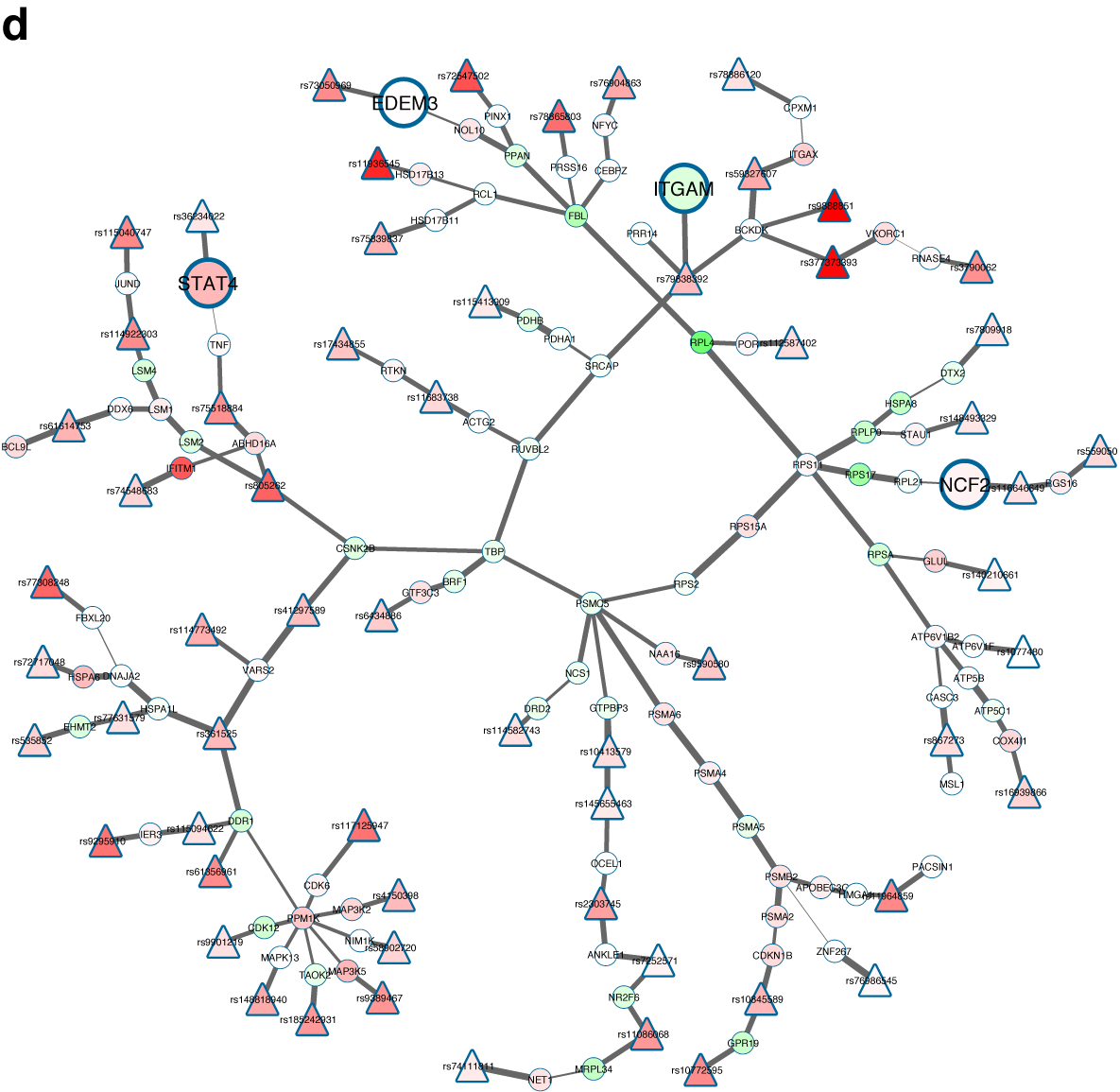
**

**
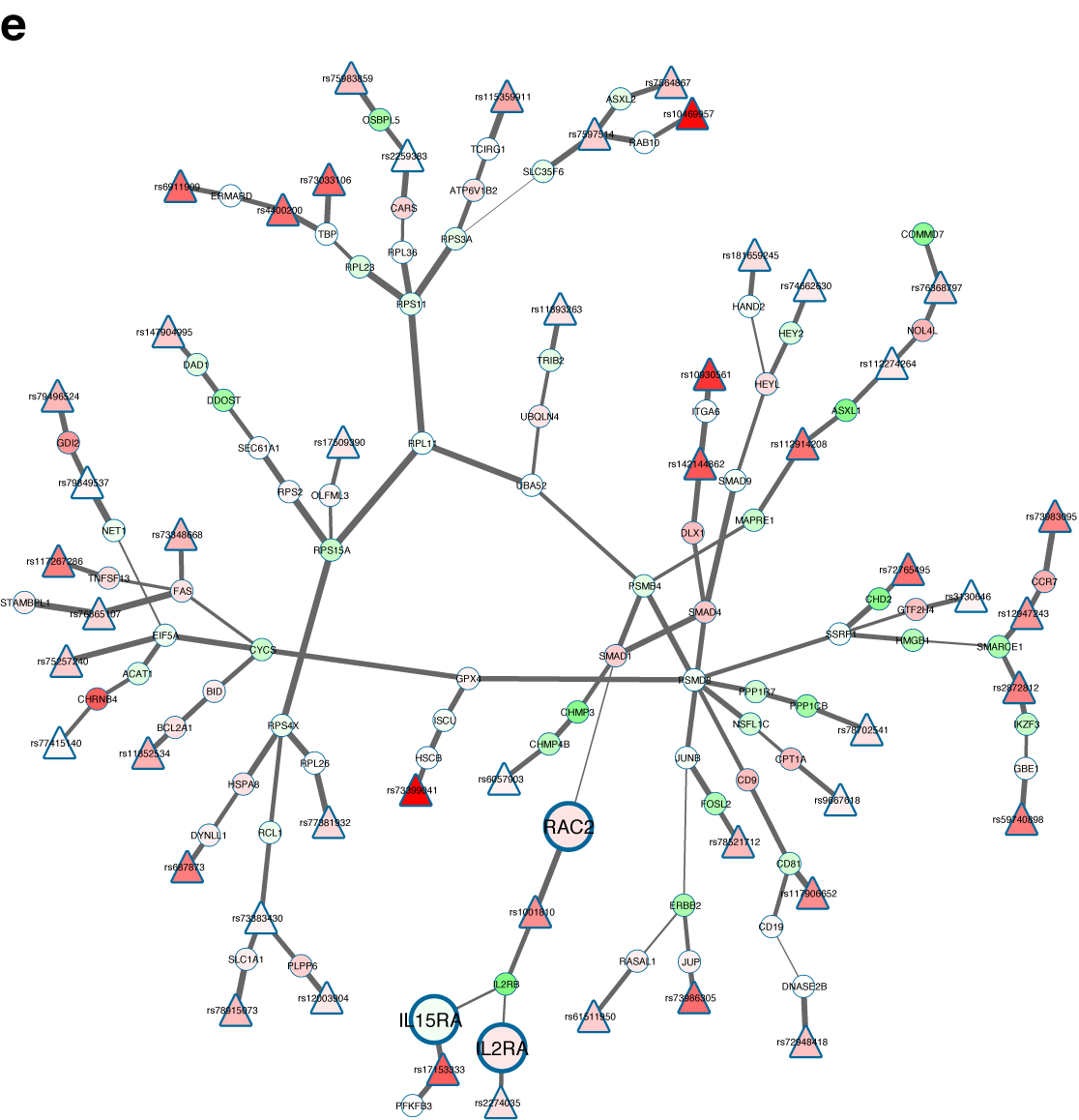
**

**
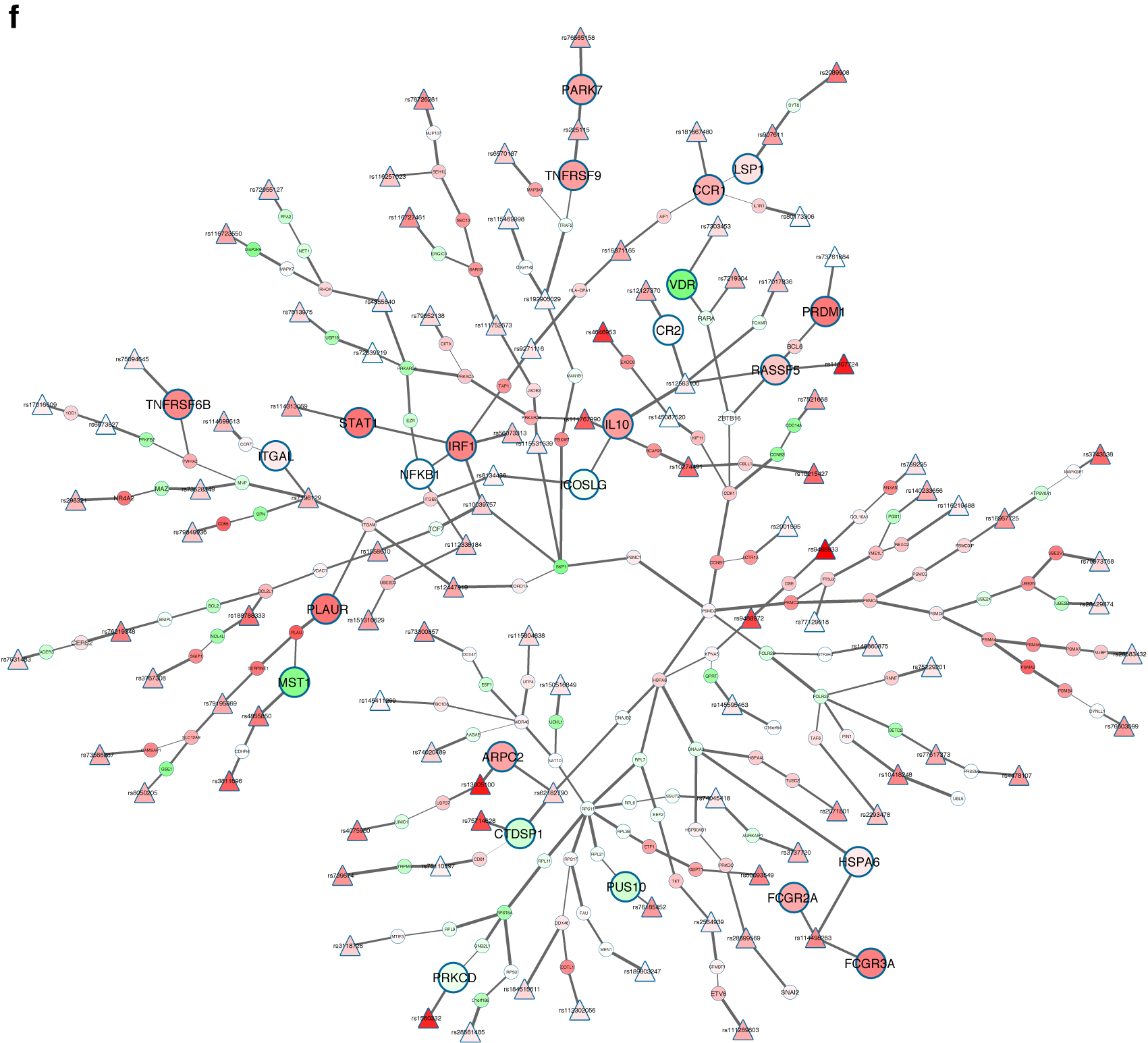
**

**Supplementary Figure 8. Performance benchmarking using functional gene interaction network obtained from the STRING database. (a)** Receiver operating characteristic curve based on known risk SNPs in promoters. **(b)** Performance evaluated using known risk SNPs in enhancers. Performance is expressed as percentile ranking on the x-axis in which each gold-standard risk SNP was ranked against all other SNPs in the same linkage equilibrium block as the gold standard SNP. Filled symbols, rank of an individual gold-standard SNP by a given method. vertical lines, median rank of the full set of gold-standard SNPs by a given method. SNP IDs and associated diseases are shown on the right. SLE, systemic lupus erythematosus; PSO, psoriasis; CRC, colorectal cancer; PRC, prostate cancer; RA, rheumatoid arthritis; OBE, obesity; MI, myocardial infarction; BRC, breast cancer; COPD, chronic obstructive pulmonary disease; SZA, schizophrenia; CAD, coronary artery disease; NBL, neuroblastoma.

**
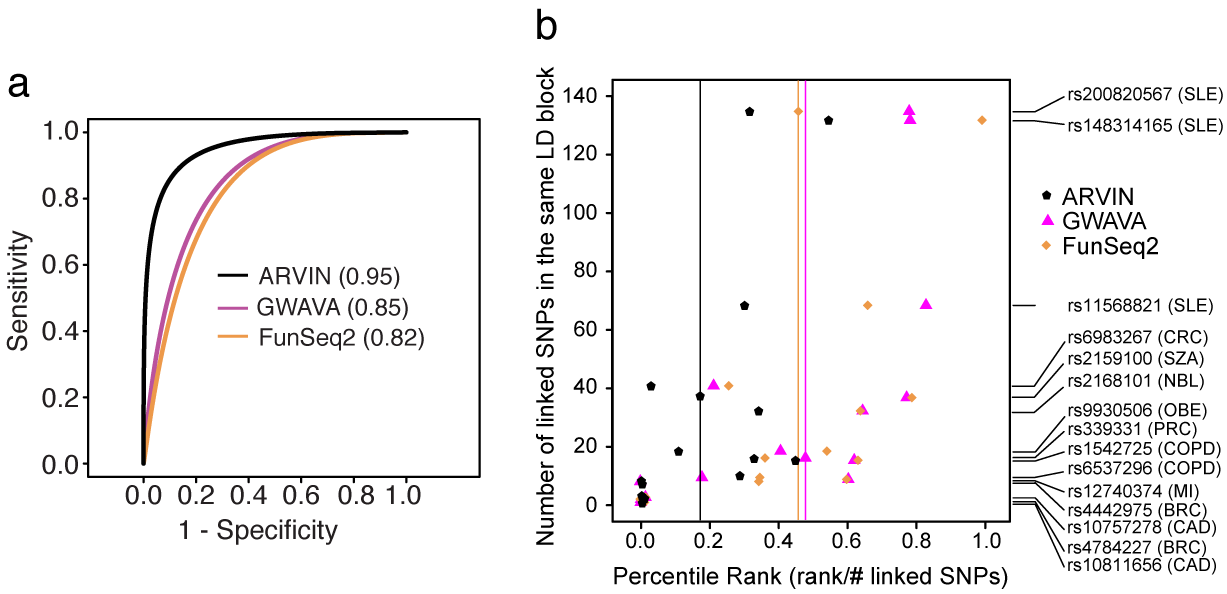
**

**Supplementary Figure 9. Performance benchmarking using transcriptional regulatory networks constructed using tissue-specific enhancer-promoter interactions derived from FANTOM5 data**. **(a)** Receiver operating characteristic curve based on known risk SNPs in promoters. **(b)** Performance evaluated using known risk SNPs in enhancers. Performance is expressed as percentile ranking on the x-axis in which each gold-standard risk SNP was ranked against all other SNPs in the same linkage equilibrium block as the gold standard SNP. Filled symbols, rank of an individual gold-standard SNP by a given method. vertical lines, median rank of the full set of gold-standard SNPs by a given method. SNP IDs and associated diseases are shown on the right. SLE, systemic lupus erythematosus; PSO, psoriasis; BRC, breast cancer; CAD, coronary artery disease. Only six of the thirteen gold-standard eSNPs have enhancer-promoter interactions based on the FANTOM5 data.


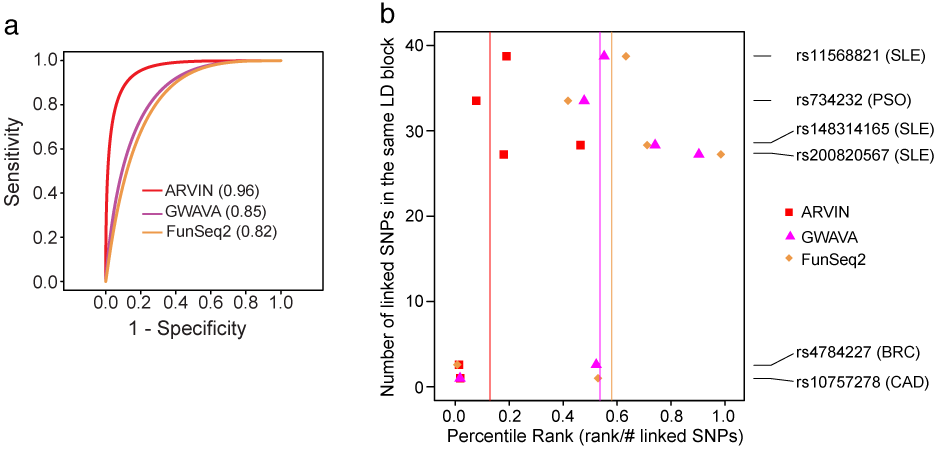


**Supplementary Table 1. Summary of data sources used for constructing tissue/cell type specific enhancer-promoter networks.** GSM*, accession IDs for NCBI GEO (Gene Expression Omnibus) database; E-MTAB*, accession IDs for EBI (European Bioinformatics Institute) Array Express database; GTEx*, Genotype-Tissue Expression Project tissue IDs used in Supplementary Table 8; HCAEC*, accession IDs for International Human Epigenome Consortium data portal; ERX*, accession IDs for European Nucleotide Archive; Reference 1, Supplementary data portal of the study by Farh *et al.* ^1^.

| **Tissue/Cell** | **Related Disease** | **Input** | **H3K4me1** | **H3K4me3** | **H3K27ac** | **RNA-Seq** |
| --- | --- | --- | --- | --- | --- | --- |
| CD4+ CD25+ CD127- (T_reg_) | Crohn’s disease, multiple sclerosis, psoriasis, rheumatoid arthritis, systemic lupus erythematous, type 1 diabetes, ulcerative colitis | GSM772914 | GSM772973 | GSM772944 | GSM997233 | PMID: 25363779 [1] |
| CD4+ CD25- IL17+ (Th_17_) |  | GSM772988 | GSM772985 | GSM772986 | GSM772987 | PMID: 25363779 [1] |
| CD4+ CD25- IL17- (Th_stim_) |  | GSM772904 GSM1112784 | GSM772902 GSM997268 | GSM916071 GSM997232 | GSM772905 GSM997266 | PMID: 25363779 [1] |
| CD8+ (T_mem_) |  | GSM772874 | GSM772873 | GSM772967 | GSM772880 | E-MTAB-2319 |
| CD4+ CD45RA+ Naïve T |  | GSM772916 GSM772876 | GSM772860 GSM772869 | GSM772836 GSM772948 | GSM772835 GSM772934 | GSM669617 GSM669583 |
| CD4+ Memory T |  | GSM772881 GSM772930 | GSM772884 GSM772924 | GSM772790 GSM772925 | GSM772963 GSM772997 | GSM669584 GSM669618 |
| B cell (GM12878) |  | GSM733742 | GSM733772 | GSM733708 | GSM733771 | GSM2072356 GSM2072357 |
| CD14+ Monocyte |  | GSM1102807 | GSM1102793 | GSM1102797 | GSM1102782 | GSM1435495 |
| Colonic mucosa | Colorectal cancer | GSM621669 | GSM621670 | GSM621671 | GSM1112802 | GTEx1 |
| Rectal mucosa |  | GSM621647 GSM621677 | GSM621639 GSM621659 | GSM621643 GSM621658 | GSM1112795 GSM1112801 |  |
| Sigmoid colon |  | GSM1059456 | GSM956020 | GSM956024 | GSM915331 | GTEx2 |
| Adipose | Obesity | GSM621401 | GSM621425 | GSM621435 | GSM916066 | GTEx3 |
| Skeletal muscle |  | GSM621680 | GSM621686 | GSM621685 | GSM916064 | GTEx4 |
| Lung | Chronic obstructive pulmonary disease | GSM906417 | GSM910572 | GSM915336 | GSM906395 | GTEx5 |
| Substantia nigra | Schizophrenia | GSM772864 | GSM772898 | GSM772901 | GSM1112778 | GTEx6 |
| Anterior caudate |  | GSM669978 GSM772826 | GSM669970 GSM772830 | GSM670031 GSM772829 | GSM1112811 GSM772832 | GTEx7 |
| Hippocampus |  | GSM669971 GSM773019 GSM916037 | GSM669962 GSM773021 GSM916039 | GSM670022 GSM773022 GSM916040 | GSM1112791 GSM773020 GSM916035 | GTEx8 |
| Mid frontal lobe |  | GSM669960 GSM773010 | GSM670015 GSM773014 | GSM670016 GSM773012 | GSM1112810 GSM773015 | GTEx9 |
| Left ventricle | Myocardial Infarction | GSM908968 | GSM906404 | GSM906406 | GSM908951 | GTEx10 |
| Endothelial cell | Coronary artery disease | HCAEC-03121 HCAEC-03193 HCAEC-03228 | HCAEC-03121 HCAEC-03193 HCAEC-03228 | HCAEC-03121 HCAEC-03193  HCAEC-03228 | HCAEC-03121  HCAEC-03193  HCAEC-03228 | HCAEC-03121  HCAEC-03193 HCAEC-03228 |
| Mammary epithelial (HMEC) | Breast cancer | GSM733668 | GSM733705 | GSM733712 | GSM733660 | GSM765397 |
| LNCAP (Prostate adenocarcinoma) | Prostate cancer | GSM686947 | GSM686928 | GSM686935 | GSM686937 | GSM1902621 GSM1902622  GSM1902623 |
| SHSY5Y (Bone marrow neuroblast) | Neuroblastoma | GSM1532410 | GSM1532409 | NA | GSM1532408 | ERX583734 |

**Supplementary Table 2. References for Hi-C and ChIA-PET data used to evaluate IM-PET prediction accuracy.**

| **Experimental technology** | **Cell type** | **Reference PMID** |
| --- | --- | --- |
| Hi-C | CD34+ | 25938943 |
|  | GM12878 | 25938943, 25497547 |
|  | HELA | 25497547 |
|  | HMEC | 25497547 |
|  | HUVEC | 25497547 |
|  | IMR90 | 25497547 |
|  | K562 | 25497547 |
|  | NHEK | 25497547 |
| ChIA-PET | CD4+ T | 22270183 |
|  | GM12878 | 26686651 |
|  | HELA | 26686651 |
|  | K562 | 22265404 |
|  | MCF-7 | 22265404 |

**Supplementary Table 3. List of gene expression datasets used for differential gene expression analysis across healthy donors and patient samples for the diseases studied.**

| **Disease** | **Accession Number** | **Source**  **Tissue/Cell Types** |
| --- | --- | --- |
| Alzheimer’s Disease | GSE48350 | Hippocampus |
| Asthma | GSE43696 | Bronchial epithelial cells |
| Autism Spectrum Disorder | GSE7329 | Lymphoblastoid cells |
| Bladder cancer | GSE3167 | Bladder |
| Breast cancer | EGEOD-54002 | Mammary gland cells |
| Chronic obstructive pulmonary disease | GSE47460 | Whole lung homogenate |
| Colorectal cancer | GSE21510 | Colon |
| Coronary artery disease | GSE20686 | Whole blood |
| Crohn’s disease | GSE20881 | Sigmoid colon  Terminal colon  Ascending colon  Descending colon |
|  | GSE36807 | Intestine |
|  | GSE9686 | Colon |
| Cystic Fibrosis | GSE15568 | Epithelial cells |
| Hypercholesterolaemia | GSE6054 | Monocytes |
| Multiple sclerosis | GSE21942 | Peripheral blood mononuclear cells |
|  | GSE43592 | T cells |
|  | GSE21942 | Peripheral blood mononuclear cells |
| Myocardial infarction | GSE66360 | CD146+ circulating endothelial |
| Neuroblastoma | E-MEXP-669 | Fetal sympathetic neuroblasts |
| Obesity | GSE55200 | Subcutaneous adipose tissue |
| Parkinson Disease | GSE7621 | Substantia nigra |
| Prostate cancer | GSE55945 | Prostate |
| Psoriasis | GSE13355 | Skin |
|  | GSE52471 |  |
|  | GSE32407 |  |
|  | GSE14905 |  |
|  | GSE10500 |  |
| Rheumatoid arthritis | GSE12021 | Synovial membrane  Synovial macrophage |
|  | GSE10500 |  |
| Schizophrenia | GSE25673 | Hippocampus |
| Systemic lupus erythematous | GSE29536 | Whole blood  CD3+ T cells  CD4+ T, CD19+ B, myeloid  Monocytes  Peripheral B cells |
|  | GSE13887 |  |
|  | GSE10325 |  |
|  | GSE46907 |  |
|  | GSE30153 |  |
| Thalassemia Beta | GSE62430 | Erythroid progenitor cells |
| Type 1 diabetes | GSE9006 | Peripheral blood  mononuclear cells |
|  | GSE55098 | Peripheral blood  mononuclear cells |
| Type 2 diabetes | GSE29226 | subcutaneous adipose |
| Ulcerative colitis | GSE10191 | Colon |
|  | GSE36807 | Intestine |
|  | GSE9686 | Colon |

**Supplementary Table 4. List of known risk SNPs located in transcriptional enhancers.** Pubmed ID is provided for the validation study. LD, linkage disequilibrium.

| **Disease** | **SNP ID** | **Target Gene(s)** | **PMID of Literature**  **Reference** | **# eSNPs in the same LD block** |
| --- | --- | --- | --- | --- |
| Systemic lupus erythematosus | rs11568821 | *PDCD1* | 12402038 | 62 |
|  | rs148314165  rs200820567 | *TNFAIP3* | 24039598 | 168 |
| Colorectal cancer | rs6983267 | *cMYC* | 19561604 | 41 |
| Obesity | rs9930506 | *IRX3* | 24646999 | 15 |
| Myocardial Infarction | rs12740374 | *SORT1* | 20686566 | 2 |
| Breast cancer | rs4442975 | *IGFBP5* | 25248036 | 6 |
|  | rs4784227 | *TOX3* | 23001124 | 1 |
| Chronic obstructive pulmonary disease | rs6537296 | *HHIP* | 22140090 | 10 |
|  | rs1542725 |  |  | 13 |
| Prostate cancer | rs339331 | *RFX6* | 24390282 | 17 |
| Schizophrenia | rs2159100 | *CACNA1C* | 25453756 | 33 |
| Coronary artery disease | rs10811656 | *CDKN2B*  *CDKN2BA*  *IFNA21*  *MTAP* | 21307941 | 1 |
|  | rs10757278 |  |  | 3 |
| Neuroblastoma | rs2168101 | *LMO1* | 23348506 | 25 |

**Supplementary Table 5. Number of NHGRI GWAS Catalog SNPs associated with autoimmune diseases and enhancer SNPs (eSNPs) in the same LD blocks with the GWAS Catalog lead SNPs.**

| Disease | # GWAS lead SNPs (p<5x10^-8^) | # eSNPs in the same LD blocks with GWAS lead SNPs |
| --- | --- | --- |
| Systemic lupus erythematosus | 82 | 6,853 |
| Psoriasis | 58 | 3,520 |
| Rheumatoid arthritis | 143 | 10,346 |
| Type 1 Diabetes | 66 | 5,092 |
| Crohn’s Disease | 273 | 15,015 |
| Ulcerative Colitis | 158 | 9,814 |
| Multiple Sclerosis | 82 | 5,966 |

**Supplementary Table 6. List of eQTL tissue/cell types that are relevant to a given autoimmune disease in this study.** CRH, Crohn’s disease; MS, multiple sclerosis; PSO, psoriasis; RA, rheumatoid arthritis; SLE, systemic lupus erythematosus; T1D, type 1 diabetes; ULC ulcerative colitis.

| **Diseases** | **Tissue/Cell types** |
| --- | --- |
| CRH | Colon_Transverse, Colon_Sigmoid, Small_Intestine_Terminal_Ileum, Stomach, Esophagus_Mucosa, Esophagus_Gastroesophageal_Junction, Whole_Blood, Cells_EBV-transformed_lymphocytes |
| MS | Brain_Anterior_cingulate_cortex_BA24, Brain_Cortex, Brain_Frontal_Cortex_BA9, Brain_Nucleus_accumbens_basal_ganglia, Brain_Hippocampus, Brain_Cerebellum, Brain_Cerebellar_Hemisphere, Brain_Putamen_basal_ganglia, Brain_Caudate_basal_ganglia, Brain_Hypothalamus, Cells_EBV-transformed_lymphocytes |
| PSO | Skin_Sun_Exposed_Lower_leg, Skin_Not_Sun_Exposed_Suprapubic, Whole_Blood, Cells_EBV-transformed_lymphocytes |
| RA | Whole_Blood, Cells_EBV-transformed_lymphocytes |
| SLE | Whole_Blood, Cells_EBV-transformed_lymphocytes |
| T1D | Pancreas, Whole_Blood, Cells_EBV-transformed_lymphocytes |
| ULC | Colon_Transverse, Colon_Sigmoid, Whole_Blood, Cells_EBV-transformed_lymphocytes |

**Supplementary Table 7. List of enhancers tested with luciferase reporter assay. NC1 & NC2, negative control sequences.** The percentile rank for each enhancer is based on ARVIN score for the eSNP among all input eSNPs to ARVIN. The larger the ARVIN score the smaller the percentile rank.

| Sequence ID | Coordinate | Percentile Rank | Primer sequence for cloning |
| --- | --- | --- | --- |
| e_rs117239407 | chr12:6,534,401-6,536,401 | 1% | F: CCGAGCTCTTACGCGTACTCATAGTGGTTTTGACTCTCT  R: CCGGGCTAGCACGCGTCCGAATTTCATTTTCAAGTTGCT |
| e_rs57572958 | chr12:6,249,801-6,251,801 | 1% | F: CCGAGCTCTTACGCGTACTAAGCTTGTGGTCATTTGTC  R: CCGGGCTAGCACGCGTCCACCAGACTCAGGAGC |
| e_rs112640799 | chr2:202,021,801-202,023,801 | 1% | F: CCGAGCTCTTACGCGTATGGCTGCCTTTATTTCCTTTA  R: CCGGGCTAGCACGCGTCAGGAGTTCGAGACCAGTC |
| e_rs244683 | chr5:133,426,201-133,428,201 | 1% | F: CCGAGCTCTTACGCGTAGAAGGAGATGGCACAGAG  R: CCGGGCTAGCACGCGTATGCCTGGCTAATTTAATGGAT |
| e_rs73983095 | chr17:38,755,201-38,757,201 | 1% | F: CCGAGCTCTTACGCGTTTGCTTAACTACATAGAATCTCGG  R: CCGGGCTAGCACGCGTTATGCATGTTGGCACTGG |
| e_rs117330495 | chr11:118,561,601-118,563,601 | 1% | F: CCGAGCTCTTACGCGTTTTCACCATGTTGGCCAG  R: CCGGGCTAGCACGCGTACCAATTTAAAGGGACACCAA |
| e_rs74053809 | chr1:8,228,401-8,230,401 | 2% | F: CCGAGCTCTTACGCGTTCGCAGCCATCCATCTTC  R: CCGGGCTAGCACGCGTCGGGACAAACTGTGTACAC |
| e_rs76986545 | chr16:31,880,401-31,882,401 | 2% | F: CCGAGCTCTTACGCGTCAAAGGAGCTGAGACTGAAAC  R: CCGGGCTAGCACGCGTTTGATGTTGAAGAGGTGCAC |
| e_rs116302151 | chr6:112,349,001-112,351,001 | 2% | F: CCGAGCTCTTACGCGTAAGAAGGTAGAGTGAGGGAAA  R: CCGGGCTAGCACGCGTTTCCACGAAGACTCCATGT |
| e_rs73790131 | chr5:133,435,001-133,437,001 | 2% | F: CCGAGCTCTTACGCGTCAGCCTTTTACATTCCCAGA  R: CCGGGCTAGCACGCGTGGCCAATCCTGTGTTTCTTA |
| e_rs7161479 | chr14:35,884,601-35,886,601 | 3% | F: CCGAGCTCTTACGCGTGGCTCCTTTTCAAACCTTCAG  R: CCGGGCTAGCACGCGTGTGCAGTCGTGTGATATCTC |
| e_rs78404309 | chr10:6,124,601-6,126,601 | 3% | F: CCGAGCTCTTACGCGTCTCTTCTACTTCTCCTGCATTG  R: CCGGGCTAGCACGCGTTAAAGTTTTAATTCATCTTCCACCTA |
| e_rs11644904 | chr10:81,948,001-81,950,001 | 91% | F: CCGAGCTCTTACGCGTCCAGGAACAGGTGGCTAC  R: CCGGGCTAGCACGCGTCAGAGGAAGGTTAGAGGCAG |
| e_rs16826559 | chr1:22,414,401-22,416,401 | 92% | F: CCGAGCTCTTACGCGTTAGGGCCTAGTGCATAATTTAA  R: CCGGGCTAGCACGCGTAACACATTCTTCAGACCTCTC |
| e_rs140008683 | chr3:45,735,401-45,737,401 | 94% | F: CCGAGCTCTTACGCGTTAAATATCCTGTTAGGCTCCA  R: CCGGGCTAGCACGCGTAAAAGGACAAATACTGCCAC |
| e_rs7068933 | chr10:81,948,001-81,950,001 | 96% | F: CCGAGCTCTTACGCGTGCATGAGTTTTACCGCAGC  R: CCGGGCTAGCACGCGTGTTAGGTGTGCAGTTCAGTG |
| NC1 (Negative Control 1) | chr1:24,000-26,000 | NA | F: CCGAGCTCTTACGCGTTCCCTGACCTGAGACCA  R: CCGGGCTAGCACGCGTGTTGGTGTGGTGCGTTAT |
| NC2 (Negative Control 2) | chr1: 430,000-432,000 | NA | F: CCGAGCTCTTACGCGTGGTAAATCACCACCCTTCAA  R: CCGGGCTAGCACGCGTGCGTAGATTCCCACTCATTC |

**Supplementary Table 8. Primers for the mutagenesis of predicted eSNPs.**

| **Sequence ID** | **SNP** | **Mutation** | **Primer sequence** |
| --- | --- | --- | --- |
| e_rs117239407 | rs117239407 | G to A | F: CTCCCCATCCAGCCCACCCCA  R: AGCTGCAAGAAGCAGGATGTTG |
| e_rs57572958 | rs57572958 | G to A | F: TCTCTCTTACATAAATTCTGCAG  R: TGCTACAAATATACAAAGAAAAG |
| e_rs112640799 | rs112640799 | C to T | F: ACAGGGGCTTTCCCAGCCAGA  R: CACATGACTGTGAGCAGACAGC |
| e_rs244683 | rs244683 | G to C | F: ATTGTGAACTCGACATTGGAAG  R: GTTGTGTGAAGAACTTTCC |
| e_rs73983095 | rs73983095 | C to T | F: GGGGAGGACAAAGGTAGGGCA  R:ACCAGCTCCTCCTCTCCTC |
| e_rs117330495 | rs117330495 | C to A | F: ATTGGCCATCTTAATAATGGGGG  R: GCCAGCCCCATAGAGAGA |
| e_rs74053809 | rs74053809 | C to T | F: AAATGAGTCATAATGAGACCATTG  R: TCTTTACCCCTACAATTC |
| e_rs76986545 | rs76986545 | C to T | F: TCCTGGCTCATATGGACCCAG  R: GCAATCTCCAAGATAGCACAAAAG |
| e_rs116302151 | rs116302151 | T to C | F: AGACTACTTGCTCATTAAGACAAGGAC  R: GCAACCACCACCAGCAGA |
| e_rs73790131 | rs73790131 | T to C | F: CTTTCTCACCCTTCTCAACCACCTTCTTTTTTTAACTTTTACTTTTTG  R: GCTGCCTCCAGGAGGCCG |
| e_rs7161479 | rs7161479 | C to A | F: GGCAAATAAAATGGAGTAGGTTAG  R: ACACTGTCTTCTAAAGCATAC |
| e_rs78404309 | rs78404309 | G to A | F: GTTCTCCCCAACTCTCCTAGGAATG  R: ACTGCCAACCGTGTGCCA |
| e_rs11644904 | rs11644904 | A to T | F: GTAGGAAAGTTTGAAGCGCTCCAG  R: TGAGGCCACTACAGCTCC |
| e_rs16826559 | rs16826559 | C to G | F: TCAAACTGATGAAACTAAAGAACATG  R: TCCCAAGTAAATATGTCC |
| e_rs140008683 | rs140008683 | G to A | F: GGCAACCACCATTCTACTTTCTGTC  R: AGGGGCTGGGAGGATACA |
| e_rs7068933 | rs7068933 | G to A | F: AACAGTATGAAGATTCCTCAAAAAATTAAAC  R: TCCATAGTGGCTGCACTA |

**References**

1 Farh, K. K. *et al.* Genetic and epigenetic fine mapping of causal autoimmune disease variants. *Nature* **518**, 337-343, doi:10.1038/nature13835 (2015).
