# Supplementary material for: Identifying noncoding risk variants using disease-relevant gene regulatory networks
Source: Nat Commun. 2018 Feb 16;9:702. doi: 10.1038/s41467-018-03133-y (PMC5816022; doi:10.1038/s41467-018-03133-y)
Supplement: Supplementary file 2 — Description of Additional Supplementary Files [file 41467_2018_3133_MOESM2_ESM.pdf]

Description of Additional Supplementary Files

File Name: Supplementary Data 1
Description: List of gold standard risk SNPs and negative control SNPs located in gene promoters. AD, Alzheimer's disease; ASD, Autism spectrum disorder; AST, asthma; BLC, bladder cancer; CAD, coronary artery disease; CF, cystic fibrosis; COPD, chronic obstructive pulmonary disease; CRC, colorectal cancer; HC, hypercholesterolaemia; MI, myocardial infarction; OBE, obesity; PD, Parkinson's disease; PRC, prostate cancer; PSO, psoriasis; RA, rheumatoid arthritis; SLE, systemic lupus erythematosus; SZA, schizophrenia; T1D, Type 1 diabetes; T2D, Type 2 diabetes; TSB, thalassemia beta.

File Name: Supplementary Data 2
Description: List of selected and all features based on recursive feature elimination. We thank you and the reviewers for your time and careful consideration of our manuscript. We hope you will agree that the manuscript is now suitable for publication in Nature Communications.
